# Systemic muscle wasting and coordinated tumour response drive tumourigenesis

**DOI:** 10.1038/s41467-020-18502-9

**Published:** 2020-09-16

**Authors:** Holly Newton, Yi-Fang Wang, Laura Camplese, Joao B. Mokochinski, Holger B. Kramer, André E. X. Brown, Louise Fets, Susumu Hirabayashi

**Affiliations:** 1 Medical Research Council London Institute of Medical Sciences, Du Cane Road, London, W12 0NN UK; 2 Institute of Clinical Sciences, Faculty of Medicine, Imperial College London, Du Cane Road, London, W12 0NN UK

**Keywords:** Cancer metabolism, Cancer metabolism

## Abstract

Cancer cells demand excess nutrients to support their proliferation, but how tumours exploit extracellular amino acids during systemic metabolic perturbations remain incompletely understood. Here, we use a *Drosophila* model of high-sugar diet (HSD)-enhanced tumourigenesis to uncover a systemic host-tumour metabolic circuit that supports tumour growth. We demonstrate coordinate induction of systemic muscle wasting with tumour-autonomous Yorkie-mediated SLC36-family amino acid transporter expression as a proline-scavenging programme to drive tumourigenesis. We identify Indole-3-propionic acid as an optimal amino acid derivative to rationally target the proline-dependency of tumour growth. Insights from this whole-animal *Drosophila* model provide a powerful approach towards the identification and therapeutic exploitation of the amino acid vulnerabilities of tumourigenesis in the context of a perturbed systemic metabolic network.

## Abstract (web summary)

Cancer is a systemic disease that associates with host metabolic changes. Here, the authors show using *Drosophila*, that tumours exploit extracellular proline in response to muscle wasting, indicating that tumours induce muscle wasting as a nutrient-scavenging programme to drive tumourigenesis.

## Introduction

Cancer cells require a constant supply of metabolic intermediates to support their proliferation. To meet the biosynthetic demands associated with tumourigenesis, cancer cells actively acquire nutrients from the extracellular space^1–5^. Cancer is a systemic disease that associates with a range of host metabolic abnormalities such as obesity, insulin resistance and cancer-associated cachexia; each of which alters the host systemic nutritional environment. These changes in both nutrient composition and availability may have profound effects on cancer development and progression. However, how cancer cells sense and respond to nutritional changes in the context of organismal metabolic alterations remains an underexplored area in cancer biology.

Cancer-associated cachexia is a systemic metabolic syndrome of weight loss associated with progressive skeletal muscle wasting^6,7^. The multifactorial and heterogeneous condition of cachexia involves a complex multi-organ interplay, which has impeded its comprehensive understanding at the molecular level^8^. A series of recent studies using *Drosophila melanogaster* have shown that tumour-derived factors modulate host metabolism^9–11^. In addition, tumour-derived factors promote the release of nutrients from the tumour microenvironment to promote tumour growth^12^. Here we leverage a *Drosophila* model of high-sugar diet (HSD)-enhanced tumourigenesis and demonstrate that HSD-enhanced tumours induce SLC36-family transporter expression as a coordinated mechanism to exploit exogenous proline for tumourigenesis during systemic muscle wasting. Furthermore, we use these mechanistic insights to rationally target the proline dependency of tumours as an approach to inhibit tumour growth.

## Results

### Ras/Src-tumours promote muscle wasting in HSD

**Fig. 1:**
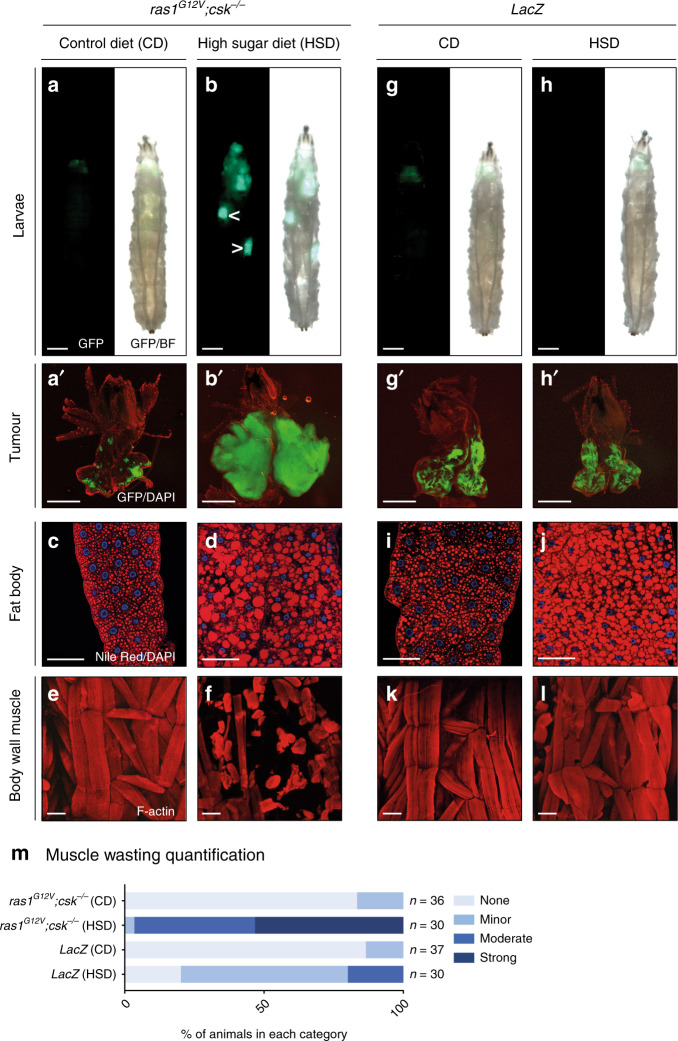
HSD-fed Ras/Src-animals exhibit systemic muscle wasting. *ras1*^*G12V*^*;csk*^*−/−*^ third-instar larvae raised on CD (**a**) or HSD (**b**). Transformed tissue is labelled with GFP (green). Secondary tumours are observed in a subset of animals (arrowheads in **b**). Scale bar, 500 μm. **a′**, **b′** Matching dissected eye epithelial tissue stained with DAPI (red). Scale bar, 250 μm. Nile Red (red) and DAPI (blue) staining of dissected fat body tissue from *ras1*^*G12V*^*;csk*^*−/−*^ third-instar larvae fed a CD (**c**) or HSD (**d**). Scale bar, 100 μm. F-actin (red) staining of dissected larval body-wall muscle tissue from *ras1*^*G12V*^*;csk*^*−/−*^ third-instar larvae raised on CD (**e**) or HSD (**f**). Scale bar, 100 μm. *LacZ* third-instar larvae raised on CD (**g**) or HSD (**h**). *LacZ*-expressing eye tissue is labelled with GFP (green). Scale bar, 500 μm. **g′**, **h′** Matching dissected eye epithelial tissue stained with DAPI (red). Scale bar, 250 μm. Nile Red (red) and DAPI (blue) staining of dissected fat body tissue from *LacZ* third-instar larvae fed a CD (**i**) or HSD (**j**). Scale bar, 100 μm. F-actin (red) staining of dissected larval body-wall muscle tissue from *ras1*^*G12V*^*;csk*^*−/*^^−^ third-instar larvae fed a CD (**k**) or HSD (**l**). Scale bar, 100 μm. **m** Matching body-wall muscle wasting quantification. *X*-axis represents the percentage of individual animals scoring in each category denoted ‘none', ‘minor', ‘moderate' or ‘strong'.

We previously reported a *Drosophila* larval model to study the systemic effects of HSD-induced obesity and insulin resistance on tumour progression^13,14^. Feeding *Drosophila* larvae an HSD led to sugar-dependent metabolic defects including accumulation of fat, systemic insulin resistance and hyperglycaemia^15^. Targeted co-activation of Ras- and Src-pathways in the *Drosophila* eye epithelia (Ras/Src-activated tumours)—by expression of an oncogenic isoform of *dRas1, ras1*^*G12V*^ and knockout of the negative regulator of Src, C-terminal src kinase (*csk*^*−/−*^)—led to the development of benign tumours in animals raised on a control diet (CD) (Fig. 1a). Comparatively, feeding *ras1*^*G12V*^*;csk*^*−/−*^ animals an HSD promoted aggressive tumour growth in the eye epithelia (Fig. 1b and Supplementary Fig. 1a), associated with secondary tumour formation (Fig. 1b, arrowheads), and led to larval lethality (Supplementary Fig. 1b).

To explore the interplay between HSD-induced obesity, tumour progression and systemic organ wasting, we assessed the peripheral tissues of *ras1*^*G12V*^*;csk*^*−/−*^ animals—including the larval fat body and body-wall skeletal muscle. Fat body lipid accumulation was elevated under HSD feeding, as assessed by the neutral lipid stain Nile Red (Fig. 1c, d). Strikingly, despite possessing an intact fat body, *ras1*^*G12V*^*;csk*^*−/*^^−^ animals raised on HSD exhibited significant skeletal muscle wasting as visualised by F-actin and myosin staining (Fig. 1e, f and Supplementary Fig. 1c, d). To quantify the severity of muscle wasting—as assessed by imaging the fourth abdominal segment of the larval body-wall muscle (Supplementary Fig. 1e)—we scored animals into one of four categories: none, minor, moderate or strong wasting (Supplementary Fig. 1f–i). While 83.3% of the *ras1*^*G12V*^*;csk*^*−/−*^ animals raised on a CD displayed intact skeletal muscle, most of the animals raised on an HSD exhibited muscle wasting of some degree (Fig. 1m). Muscle wasting was progressive; as HSD-enhanced tumourigenesis proceeded, skeletal muscle wasting increased (Supplementary Fig. 1j–n). Functional locomotion defects—as measured by video tracking analysis^16,17^—were observed in *ras1*^*G12V*^*;csk*^*−/−*^ animals raised on an HSD (Supplementary Fig. 1o, p).

Obesity and insulin resistance is an independent factor that can promote muscle wasting^18^. HSD feeding promoted lipid accumulation in tumour-free *LacZ*-animals, and also led to muscle wasting, albeit far weaker than *ras1*^*G12V*^*,csk*^*−/−*^ animals in HSD (Fig. 1g–m). These results indicate that HSD-induced obesity induces systemic muscle wasting, and the presence of tumours further promotes muscle wasting.

To examine the possibility that observed muscle wasting was due to the ecdysone-mediated metamorphic process, we used the temperature-sensitive *ecdysoneless* mutant allele (*ecd*^*1*^)^19^. Tumour-free and *ras1*^*G12V*^*;csk*^*−/−*^ animals raised on HSD, exhibited muscle wasting of similar strength, regardless of whether they lacked *ecdysoneless* (*ecd*^*−/−*^), indicating that muscle wasting occurred independently of ecdysone-mediated histolysis (Supplementary Fig. 2a–e). A blue dye feeding assay indicated that *ras1*^*G12V*^*;csk*^*−/−*^ animals continue to feed in HSD (Supplementary Fig. 2f–k) and failure to detect leakage of blue dye from the gut lumen suggested intact gut integrity (Supplementary Fig. 2l–n). The morphological changes in salivary gland and midgut that occur during metamorphosis^20^ were not observed in HSD-fed *ras1*^*G12V*^*;csk*^*−/−*^ animals (Supplementary Fig. 2o–t). Collectively, these results indicate that the metamorphic process does not account for the muscle wasting observed in *ras1*^*G12V*^*;csk*^*−/−*^ animals in HSD, and instead support a specific role for HSD feeding and tumourigenesis in the promotion of muscle wasting.

### Tumour-derived branchless mediates muscle wasting

**Fig. 2:**
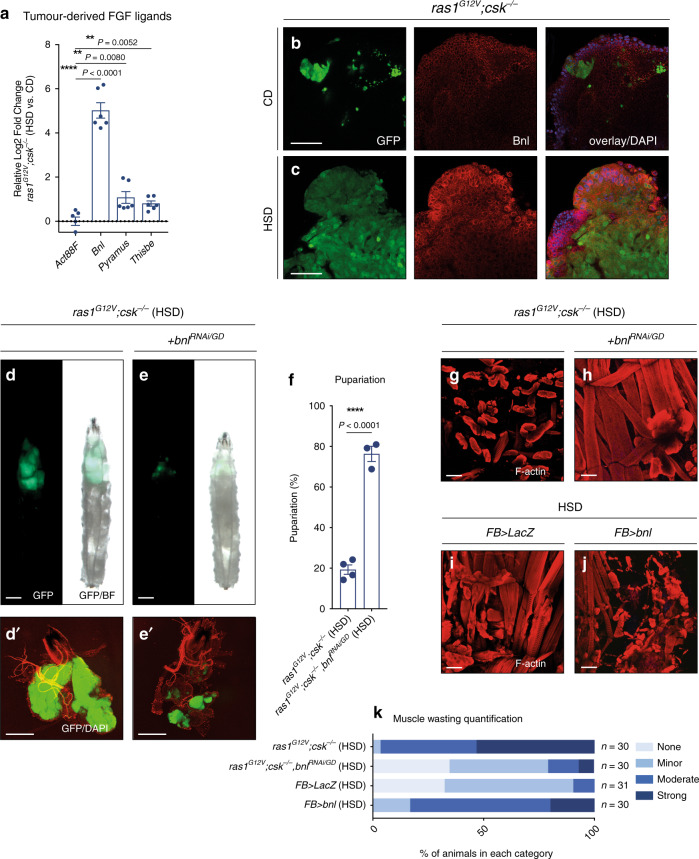
Tumour-derived branchless mediates muscle wasting and tumour growth. **a** Relative log2fold change of *Drosophila* fibroblast growth factors, *branchless* (*bnl*), *pyramus* and *thisbe* in dissected tumour tissue from *ras1*^*G12V*^*;csk*^*−/−*^ animals raised on HSD compared to animals raised on CD, as determined by qPCR. Samples are normalised to *Act88F*. Results are shown as mean ± SEM. Data from *n* = 6 biologically independent samples. Data were analysed by two-tailed unpaired Student’s *t* test. Asterisks indicate statistically significant difference (**P < 0.01; ****P < 0.0001). Anti-Bnl staining (red) of dissected tumour tissue from *ras1*^*G12V*^*;csk*^*−/−*^ animals raised on CD (**b**) or HSD (**c**) with DAPI (blue). Scale bar, 40 μm. Third-instar larvae from *ras1*^*G12V*^*;csk*^*−/−*^ (**d**), and *ras1*^*G12V*^*;csk*^*−/−*^*,bnl*^*RNAi*/GD^ (**e**). Scale bar, 500 μm. **d′**, **e′** Matching dissected eye epithelial tissue stained with DAPI (red). Scale bar, 250 μm. **f** Pupariation percentage of *ras1*^*G12V*^*;csk*^*−/−*^, and *ras1*^*G12V*^*;csk*^*−/−*^*,bnl*^*RNAi*/GD^ animals raised on HSD. Results are shown as mean ± SEM. Data from total *n* = 120 (*ras1*^*G12V*^*;csk*^*−/−*^), and *n* = 125 (*ras1*^*G12V*^*;csk*^*−/−*^*,bnl*^*RNAi*/GD^) from four independent experiments. Data were analysed by two-tailed unpaired Student’s *t* test. Asterisks indicate statistically significant difference (****P < 0.0001). F-actin (red) staining of dissected larval body-wall muscle tissue from *ras1*^*G12V*^*;csk*^*−/−*^ (**g**) or *ras1*^*G12V*^*;csk*^*−/−*^*,bnl*^*RNAi*/GD^ (**h**) third-instar larvae raised on HSD. Scale bar, 100 μm. F-actin (red) staining of dissected larval body-wall muscle tissue from *FB* > *LacZ* (**i**) or *FB* > *bnl* (**j**) third-instar larvae raised on HSD. Scale bar, 100 μm. **k** Matching body-wall muscle wasting quantification.

We next set out to identify tumour-derived factors that promote muscle wasting in HSD. Through genome-wide transcriptional profiling analysis of dissected tumour tissue from *ras1*^*G12V*^*;csk*^*−/−*^ animals raised on CD and HSD (Supplementary Fig. 3a), we identified *branchless* (*bnl*), a *Drosophila* fibroblast growth factor (FGF) ligand as one of the most highly upregulated secreted factors in *ras1*^*G12V*^*;csk*^*−/−*^ tumours in animals raised on an HSD (upregulated 5.0-log2fold) (Fig. 2a and Supplementary Fig. 3b). Among the three known *Drosophila* FGF ligands (Bnl, Pyramus and Thisbe), *bnl* was the most highly elevated FGF ligand in *ras1*^*G12V*^*;csk*^*−/−*^ tumours in HSD (Fig. 2a). Our previous study demonstrated that Bnl protein expression was elevated in the secondary tumours associated with tracheal branches^13^; we now demonstrate that Bnl protein expression is strongly upregulated in *ras1*^*G12V*^*;csk*^*−/−*^ primary tumours, but not in the wild-type (*LacZ*) eye discs of animals fed an HSD (Fig. 2b, c and Supplementary Fig. 3c–e).

Reducing *bnl* expression in Ras/Src-activated tumours (*ras1*^*G12V*^*;csk*^*−/−*^*,bnl*^*RNAi*^) attenuated muscle wasting in animals raised on an HSD (Fig. 2g, h, k and Supplementary Fig. 3f, g, q), indicating that tumour-derived Bnl is required for muscle wasting. Tumour-derived factors recently implicated in cachexia-like organ wasting include ImpL2 and Pvf1^9–11^. However, reducing the levels of either ImpL2 (*ras1*^*G12V*^*;csk*^*−/−*^*,ImpL2*^*RNAi*^) or Pvf1 (*ras1*^*G12V*^*,pvf1*^*RNAi*^*;csk*^*−/−*^) failed to rescue muscle wasting as efficiently as *bnl* in our HSD-enhanced Ras/Src-tumour model (Supplementary Fig. 3h–k, q). Similarly, reducing the expression of Pvf2 (*ras1*^*G12V*^*;csk*^*−/−*^*,pvf2*^*RNAi*^) did not rescue muscle wasting (Supplementary Fig. 3l, m, q). Ectopic expression of *bnl* from the fat body promoted muscle wasting in HSD, indicating that systemic Bnl promotes muscle wasting (Fig. 2i–k). Altogether, these results demonstrate Bnl as a tumour-derived factor that contributes to muscle wasting in Ras/Src-animals in HSD.

Importantly, reducing *bnl* expression in HSD-enhanced Ras/Src-tumours (*ras1*^*G12V*^*;csk*^*−/−*^*,bnl*^*RNAi*^) not only attenuated systemic muscle wasting but also suppressed tumour growth and larval lethality (Fig. 2d–f and Supplementary Fig. 3p). Tumour autonomous Bnl and its receptor Breathless (Btl) signalling has been implicated in tumourigenesis^21^. However, reducing the levels of *btl* in Ras/Src-activated tumours (*ras1*^*G12V*^*,btl*^*RNAi*^*;csk*^*−/−*^) had minimal effect on primary tumour size or muscle wasting, indicating that tumour autonomous reduction of Bnl–Btl signalling was not responsible for the suppression of tumour growth in *ras1*^*G12V*^*;csk*^*−/−*^*,bnl*^*RNAi*^ animals (Supplementary Fig. 3n–q). Together, these results suggest that systemic muscle wasting has a functional effect on tumour growth.

### Muscle wasting associates with circulating proline

**Fig. 3:**
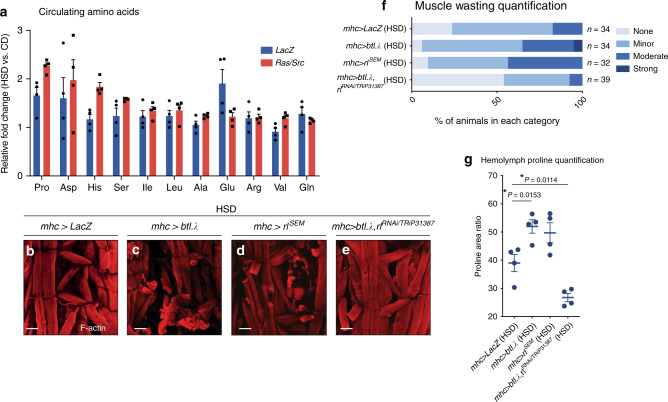
Muscle-specific Btl-dERK signalling promotes muscle wasting in HSD. **a** Circulating haemolymph levels of upregulated amino acids in *LacZ* (blue bars) and *ras1*^*G12V*^*;csk*^*−/−*^ (red bars) animals raised on HSD compared to animals raised on CD. Results are shown as mean ± SEM. Data from *n* = 4 biologically independent samples. F-actin (red) staining of dissected larval body-wall muscle tissue from *mhc* > *LacZ* (**b**), *mhc* > *btl.λ* (**c**), *mhc* > *rl*^*SEM*^ (**d**), and *mhc* > *btl.λ, rl*^*RNAi/TRiP31387*^ (**e**) third-instar larvae raised on HSD. Scale bar, 100 μm. **f** Matching body-wall muscle wasting quantification. **g** Matching haemolymph proline quantification. Results are shown as mean ± SEM. Data from *n* = 4 biologically independent samples. Data were analysed by two-tailed unpaired Student’s *t* test. Asterisks indicate statistically significant difference (*P < 0.05).

A consequence of skeletal muscle wasting is protein degradation and subsequent release of free amino acids into the circulation. Through targeted metabolomic analysis, we measured an increase in circulating levels of amino acids in the haemolymph of tumour-free *LacZ*-animals raised on an HSD relative to CD (Fig. 3a and Supplementary Fig. 4a). Consistent with enhanced muscle wasting in the presence of tumours (Fig. 1f, l, m), circulating amino acids were further elevated in *ras1*^*G12V*^*;csk*^*−/−*^ animals raised on an HSD (Fig. 3a and Supplementary Fig. 4a). Together with increased circulating 3-methylhistidine, a biomarker for muscle atrophy^22^ (Supplementary Fig. 4b), these results indicate that in tumour-bearing animals raised on an HSD, circulating levels of amino acids are further elevated.

Ectopic expression of *bnl* from the fat body not only promoted muscle wasting in HSD (Fig. 2i–k), but also led to elevated proline—the most elevated amino acid in the circulation—in the haemolymph (Supplementary Fig. 4c). Furthermore, reducing *bnl* expression in HSD-enhanced Ras/Src-tumours (*ras1*^*G12V*^;*csk*^*−/−*^*,bnl*^*RNAi*^) not only attenuated systemic muscle wasting (Fig. 2g, h and Supplementary Fig. 3f, g, p), but also reduced circulating levels of proline (Supplementary Fig. 4d). These results support the association of ectopic- or tumour-derived Bnl with muscle wasting and circulating proline levels.

We performed a genome-wide transcriptional profiling analysis of dissected body-wall muscle from *ras1*^*G12V*^*;csk*^*−/−*^ animals raised on CD and HSD (Supplementary Fig. 4e). Gene set enrichment analysis (GSEA) revealed a progressive deregulation of muscle structure organisation in *ras1*^*G12V*^*;csk*^*−/−*^ animals in HSD (Supplementary Fig. 4f). Bnl binds to its only receptor *breathless* (*btl*) and activates downstream Ras-ERK signalling^23^. Elevated ERK signalling promotes muscle wasting in adult *Drosophila* and in *C. elegans*^11,24^. GSEA analysis of muscle RNA-sequencing data revealed an increase in ERK-signalling pathway activity in *ras1*^*G12V*^*;csk*^*−/−*^ animals in HSD, which correlated with increased phospho-ERK (pERK) staining (Supplementary Fig. 4g–i). Muscle-specific expression of an active form of btl (*btl.λ*) or ERK (*rl*^*SEM*^) promoted muscle wasting and circulating proline levels (Fig. 3b–d, f, g). Reducing the expression of ERK in this setting (*mhc* > *btl.λ*, *rl*^*RNAi*^) rescued muscle wasting and proline levels (Fig. 3e–g and Supplementary Fig. 4j–l). Overall, these analyses demonstrate that muscle-specific activation of btl-ERK signalling promotes muscle wasting which correlates with circulating proline levels. Although our data suggest that tumour-derived bnl may promote muscle wasting through btl in the muscle, tumour-derived bnl may also mediate muscle wasting indirectly through other mechanisms.

### SLC36-family transporters are required for tumour growth

**Fig. 4:**
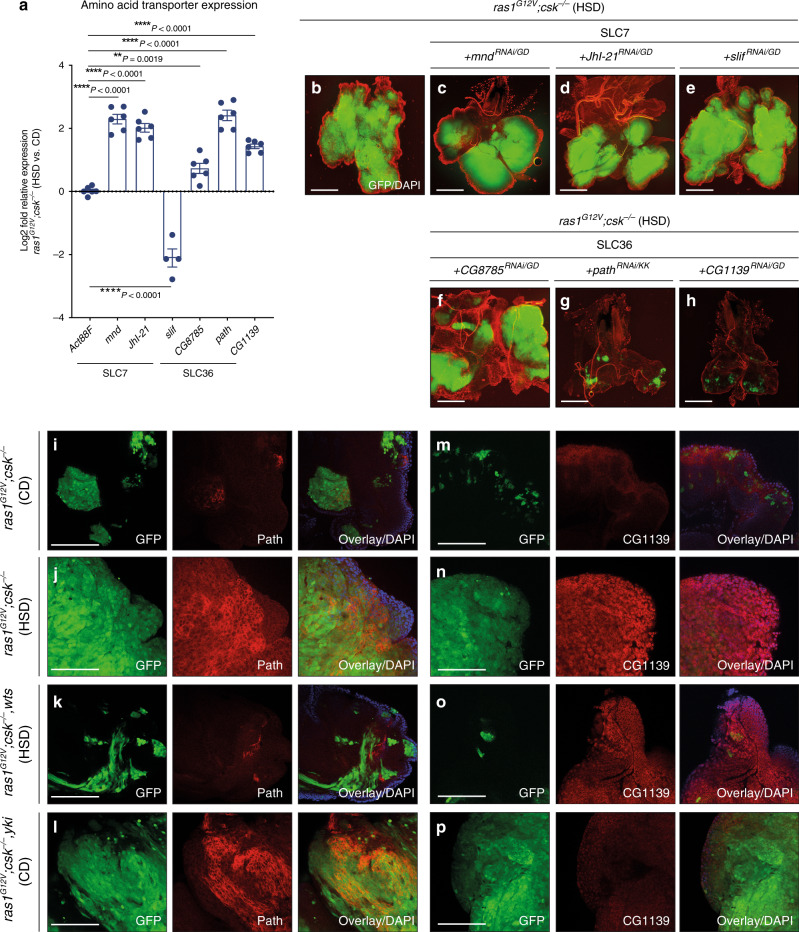
Amino acid transporter Path is required for HSD-enhanced Ras/Src-tumour growth. **a** Relative log2fold change of SLC7- and SLC36-family amino acid transporters in dissected tumour tissue from *ras1*^*G12V*^*;csk*^*−/−*^ animals raised on HSD compared to animals raised on CD, as determined by qPCR. Samples are normalised to *Act88F*. Results are shown as mean ± SEM. Data from *n* = 6 (*Act88F*), *n* = 6 (*mnd*), *n* = 6 (*JhI-21*), *n* = 4 (*slif*), *n* = 6 (*CG8785*), *n* = 6 (*path*) and *n* = 6 (*CG1139*) biologically independent samples. Data were analysed by two-tailed unpaired Student’s *t* test. Asterisks indicate statistically significant difference (**P < 0.01; ****P < 0.0001). Dissected eye epithelial tissue stained with DAPI (red) from *ras1*^*G12V*^*;csk*^*−/−*^ animals raised on HSD (**b**), with *mnd*^*RNAi/GD*^ (**c**)*, JhI-21*^*RNAi/GD*^ (**d**)*, slif*^*RNAi*/GD^ (**e**)*, CG8785*^*RNAi/GD*^ (**f**)*, path*^*RNAi/KK*^ (**g**) and *CG1139*^*RNAi/GD*^ (**h**). Scale bar, 250 μm. Anti-Path staining (red) of dissected tumour tissue from *ras1*^*G12V*^*;csk*^*−/−*^ animals raised on CD (**i**), *ras1*^*G12V*^*;csk*^*−/−*^ animals raised on HSD (**j**), *ras1*^*G12V*^*;csk*^*−/−*^*,wts* animals raised on HSD (**k**) and *ras1*^*G12V*^*;csk*^*−/−*^*,yki* animals raised on CD (**l**) with DAPI (blue). Scale bar, 100 μm. Anti-CG1139 staining (red) of dissected tumour tissue from *ras1*^*G12V*^*;csk*^*−/−*^animals raised on CD (**m**), *ras1*^*G12V*^*;csk*^*−/−*^ animals raised on HSD (**n**) and *ras1*^*G12V*^*;csk*^*−/−*^*,wts* animals raised on HSD (**o**), and *ras1*^*G12V*^*;csk*^*−/−*^*,yki* animals raised on CD (**p**) with DAPI (blue). Scale bar, 100 μm.

Since amino acids are one of the largest contributors to increased cell mass in proliferating cells^1^, our observations suggested muscle wasting as a source of circulating amino acids for tumour progression. To explore candidates that could be responsible for tumour-specific amino acid utilisation, we focused on amino acid transporters. Through RNA-sequencing analysis of tumour tissue from *ras1*^*G12V*^*;csk*^*−/−*^ animals raised on CD and HSD, we characterised the expression profile of amino acid transporters across the SLC1, SLC7, SLC36 and SLC38 families (Supplementary Fig. 5a). Subsequent qPCR validation confirmed that amino acid transporters of the SLC7 family (*minidiscs (mnd)* and *JhI-21*) and SLC36 family (*CG8785, pathetic (path)* and *CG1139*) were upregulated in tumours of *ras1*^*G12V*^*;csk*^*−/−*^ animals raised on an HSD compared to CD (Fig. 4a). Of note, expression levels of *Slimfast* (*slif*)—another SLC7 family member and an amino acid transporter previously implicated in tumour-specific usage of amino acids^12,25^—were downregulated in Ras/Src-tumours in HSD (Fig. 4a).

Reducing the expression of *mnd (ras1*^*G12V*^*;csk*^*−/−*^*,mnd*^*RNAi*^*), JhI-21 (ras1*^*G12V*^*;csk*^*−/−*^*,JhI-21*^*RNAi*^*), slif (ras1*^*G12V*^*;csk*^*−/−*^*,slif*^*RNAi*^*)* or *CG8785* (*ras1*^*G12V*^*;csk*^*−/−*^*, CG8785*^*RNAi*^) had no effect on primary tumour size (Fig. 4b–f and Supplementary Fig. 5g). Strikingly, reducing expression of *path* (*ras1*^*G12V*^*,path*^*RNAi*^;*csk*^*−/−*^) or *CG1139* (*ras1*^*G12V*^*;csk*^*−/−*^*,CG1139*^*RNAi*^) almost completely suppressed HSD-mediated Ras/Src-tumour growth (Fig. 4g, h and Supplementary Fig. 5b, c, g). Importantly, reduction in *path* or *CG1139* levels had minimal effect on tumour growth in *ras1*^*G12V*^*;csk*^*−/−*^ animals fed a CD (Supplementary Fig. 5d–g), indicating that neither is required for benign tumour growth. Altogether, these results demonstrate that Path and CG1139 are specifically required for HSD-mediated enhancement of Ras/Src-tumour growth.

We confirmed elevation of Path and CG1139 protein levels in Ras/Src-tumours in HSD using the Path- and CG1139-specific antibodies^26^ (Fig. 4i, j, m, n and Supplementary Fig. 5h–k). *Path* has been previously identified as one of the growth-regulatory factors under the control of the Hippo signalling pathway downstream transcriptional co-activator Yorkie (Yki)^27^. Overexpression of Warts (Wts)—and therefore inhibition of Yki-activity—in Ras/Src-activated cells (*ras1*^*G12V*^*;csk*^*−/−*^*,wts*) led to decreased Path protein but not CG1139 levels in animals fed an HSD (Fig. 4k, o). Conversely, the expression of Yki in Ras/Src-activated cells (*ras1*^*G12V*^*;csk*^*−/−*^*,Yki*) led to increased *path* transcript and Path protein expression in animals raised on CD, but not CG1139 (Fig. 4l, p and Supplementary Fig. 5j–l). While Path was elevated specifically in Ras/Src-activated tumours in HSD (Fig. 4j, k and Supplementary Fig. 5h), the elevation of CG1139 was not specific to tumours, but was rather dependent on HSD (Fig. 4n, o and Supplementary Fig. 5i). Together with our previous study demonstrating that HSD-enhanced Ras/Src-tumours activate Yki^14^, we conclude that the Hippo signalling downstream effector Yki mediates *path* expression in *ras1*^*G12V*^*;csk*^*−/−*^ animals raised on an HSD.

### Proline promotes tumour growth via Path-Tor-S6K signalling

**Fig. 5:**
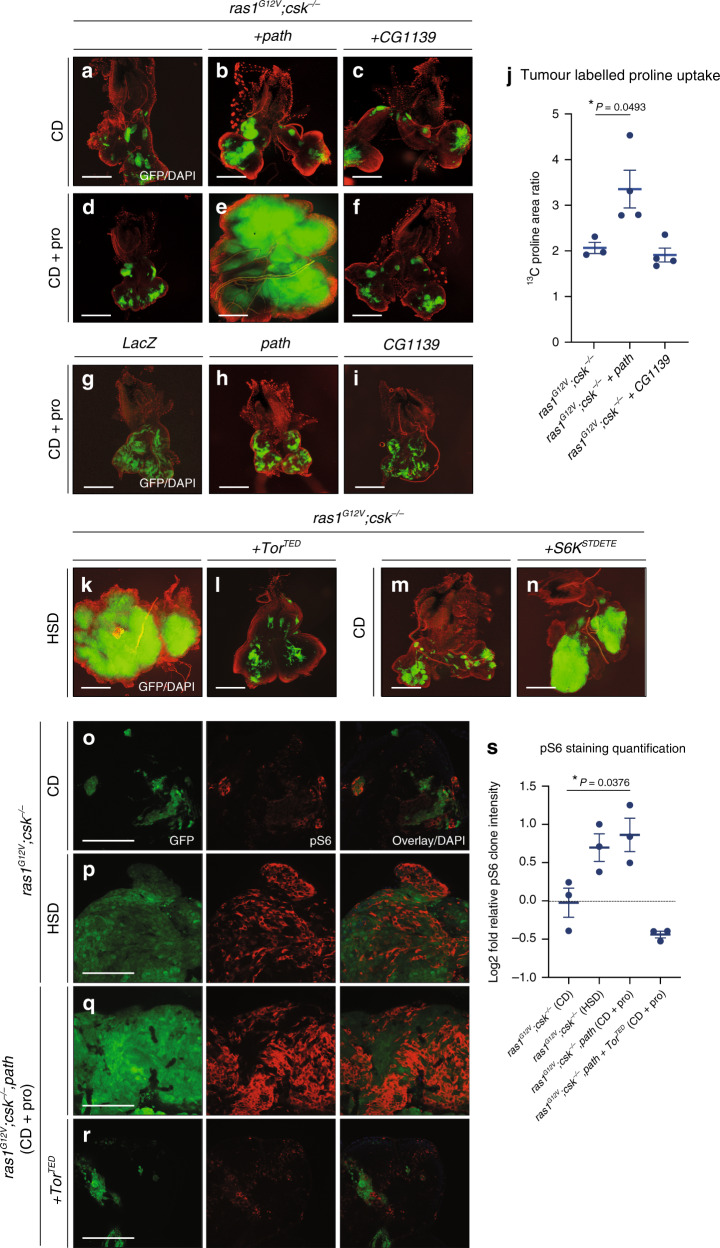
Proline promotes Ras/Src-tumour growth through amino acid transporter Path. Dissected eye epithelial tissue stained with DAPI (red) from *ras1*^*G12V*^*;csk*^*−/−*^ (**a**), *ras1*^*G12V*^*;csk*^*−/−*^*,path* (**b**) and *ras1*^*G12V*^*,CG1139;csk*^*−/−*^ (**c**) animals raised on CD. Scale bar, 250 μm. Dissected eye epithelial tissue stained with DAPI (red) from *ras1*^*G12V*^*;csk*^*−/−*^ (**d**), *ras1*^*G12V*^*;csk*^*−/−*^*,path* (**e**) and *ras1*^*G12V*^*,CG1139;csk*^*−/−*^ (**f**) animals raised on CD supplemented with 100 mM L-proline. Scale bar, 250 μm. Dissected eye epithelial tissue stained with DAPI (red) from *LacZ* (**g**), *Path* (**h**) and *CG1139* (**i**) animals raised on CD supplemented with 100 mM L-proline. Scale bar, 250 μm. **j** Stable-isotope labelled proline uptake in tumours from *ras1*^*G12V*^*;csk*^*−/−*^ animals, *ras1*^*G12V*^*;csk*^*−/−*^*,path* animals and *ras1*^*G12V*^*,CG1139;csk*^*−/−*^ animals raised on CD supplemented with 100 mM ^13^C-labelled L-proline. Results are shown as mean ± SEM. Data from *n* = 3 (*ras1*^*G12V*^*;csk*^*−/−*^), *n* = 4 (*ras1*^*G12V*^*;csk*^*−/−*^*,path*) and *n* = 4 (*ras1*^*G12V*^*,CG1139;csk*^*−/−*^) biologically independent samples. Data were analysed by two-tailed unpaired Student’s *t* test. Asterisks indicate statistically significant difference (*P < 0.05). Dissected eye epithelial tissue stained with DAPI (red) from *ras1*^*G12V*^*;csk*^*−/−*^ (**k**) and *ras1*^*G12V*^*,Tor*^*TED*^*;csk*^*−/−*^ (**l**) animals raised on HSD. Scale bar, 250 μm. Dissected eye epithelial tissue stained with DAPI (red) from *ras1*^*G12V*^*;csk*^*−/−*^ (**m**) and *ras1*^*G12V*^*; S6K*^*STDETE*^*,csk*^*−/−*^ (**n**) animals raised on CD. Scale bar, 250 μm. Anti-phospho-S6 (pS6) staining (red) of dissected tumour tissue from *ras1*^*G12V*^*;csk*^*−/−*^ animals raised on CD (**o**), *ras1*^*G12V*^*;csk*^*−/−*^ animals raised on HSD (**p**), *ras1*^*G12V*^*;csk*^*−/−*^*,path* animals raised on CD supplemented with 100 mM L-proline (**q**) and *ras1*^*G12V*^*,Tor*^*TED*^*;csk*^*−/−*^*,path* animals raised on CD supplemented with 100 mM L-proline (**r**) with DAPI (blue). Scale bar, 100 μm. **s** Matching phospho-S6 (pS6) staining quantification. Results are shown as mean ± SEM. Data from *n* = 3 biologically independent samples. Data were analysed by two-tailed unpaired Student’s *t* test. Asterisks indicate statistically significant difference (*P < 0.05).

Mammalian SLC36-family amino acid transporters including SLC36A1 and SLC36A2 transport alanine, glycine and proline^28^. Proline was the most highly increased amino acid in the haemolymph of *ras1*^*G12V*^*;csk*^*−/−*^ animals in HSD (Fig. 3a and Supplementary Fig. 4a). Overexpression of Path (*ras1*^*G12V*^*;csk*^*−/−*^*,path*) or CG1139 (*ras1*^*G12V*^*,CG1139;csk*^*−/−*^) had a minor, yet insignificant effect on tumour growth in Ras/Src-activated cells on CD (Fig. 5a–c and Supplementary Fig. 6a).

To mimic the elevation of circulating proline levels under conditions of muscle wasting, we carried out dietary supplementation of proline. Feeding animals CD supplemented with 100 mM L-proline had minimal effect on tumour growth of *ras1*^*G12V*^*;csk*^*−/−*^ animals in CD (Fig. 5d and Supplementary Fig. 6a). Intriguingly, however, feeding proline in *ras1*^*G12V*^*;csk*^*−/−*^*,path* animals strongly promoted tumour growth in CD (Fig. 5e and Supplementary Fig. 6a). In striking contrast, proline feeding in *ras1*^*G12V*^*,CG1139;csk*^*−/−*^ animals had no effect on tumour growth (Fig. 5f and Supplementary Fig. 6a). Of note, feeding proline in animals with path or CG1139 overexpressing clones had a negligible effect on eye tissue growth (Fig. 5g–i and Supplementary Fig. 6b), suggesting that proline-Path-mediated enhancement of tissue growth requires an oncogenic background. Feeding the biologically inert isomer, D-proline had no tumour-promoting effect in both *ras1*^*G12V*^*;csk*^*−/−*^ and *ras1*^*G12V*^*;csk*^*−/−*^*,path* animals (Supplementary Fig. 6e, h, k), thereby indicating that tumour response is specific to L-proline. Feeding animals CD supplemented with 100 mM of stable isotope of L-proline (^13^C^15^N-proline) led to increased proline in *ras1*^*G12V*^*;csk*^*−/−*^*,path* tumours but not in *ras1*^*G12V*^*,CG1139;csk*^*−/−*^ tumours (Fig. 5j), revealing that Path mediates tumour uptake of circulating proline. While feeding excess L-glycine marginally promoted growth of *ras1*^*G12V*^*;csk*^*−/−*^*,path* tumours, L-alanine had no growth-promoting effect in *ras1*^*G12V*^*;csk*^*−/−*^, *ras1*^*G12V*^*;csk*^*−/−*^*,path* or *ras1*^*G12V*^*,CG1139;csk*^*−/−*^ animals (Supplementary Fig. 6c, d, f, g, i–k). Altogether, our data highlight the proline vulnerability of HSD-enhanced Ras/Src-tumours and uncover modulation of amino acid transporter repertoire as a strategy to meet the nutrient requirements of tumourigenesis.

Tor-S6K signalling is implicated downstream of SLC36 transporters^29,30^. Expression of a dominant-negative isoform of Tor in Ras/Src-activated cells (*ras1*^*G12V*^*,Tor*^*TED*^*;csk*^*−/−*^) suppressed tumour growth in HSD (Fig. 5k, l and Supplementary Fig. 6l). Conversely, expression of an active isoform S6K in Ras/Src-activated cells (*ras1*^*G12V*^*;csk*^*−/−*^*,S6K*^*STDETE*^) promoted tumour growth in CD (Fig. 5m, n and Supplementary Fig. 6l). Increased phospho-specific S6 (pS6) antibody^31^ staining was observed in *ras1*^*G12V*^*;csk*^*−/−*^ tumours of animals in HSD compared to CD (Fig. 5o, p, s). Consistently, increased phospho-S6 staining was observed autonomously within *ras1*^*G12V*^*;csk*^*−/−*^*,path* tumours but not in *ras1*^*G12V*^*,Tor*^*TED*^*csk*^*−/−*^*,path* tumours of animals raised on a proline-supplemented diet (Fig. 5q–s). These results demonstrate that proline-Path promotes Tor-S6K signalling.

We next examined the link between Path-mediated Ras/Src-tumour growth and muscle wasting. Feeding proline in *ras1*^*G12V*^;*csk*^*−/−*^*,path* animals not only promoted tumour growth (Fig. 5e), but also led to elevated tumour expression of Bnl (Supplementary Fig. 7a, b, h) and muscle wasting in animals raised on CD (Supplementary Fig. 7c–e). In addition, *ras1*^*G12V*^;*csk*^*−/−*^*, S6K*^*STDETE*^ tumours promoted expression of Bnl, suggesting that increased Path-Tor-S6K signalling promotes *bnl* expression (Supplementary Fig. 7f–h). Together these results demonstrate that proline-Path-mediated tumour growth promotes Bnl expression and muscle wasting. Overall, our data implicate the coordinate induction of tumour-autonomous amino acid transporter expression, coupled with systemic muscle wasting, as sufficient to establish a feed-forward host-tumour circuit to drive tumour growth.

### Targeting proline vulnerability of tumours

**Fig. 6:**
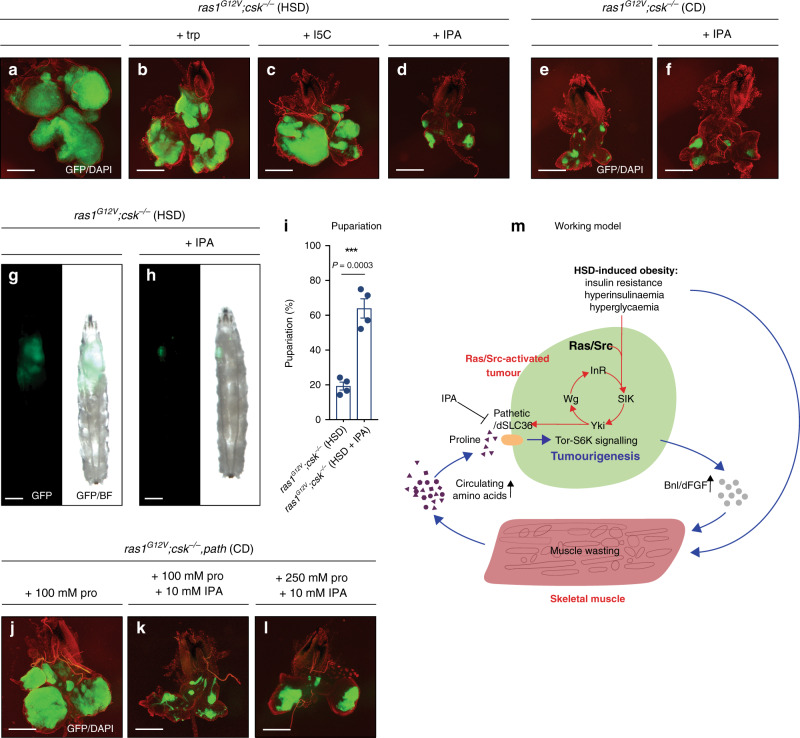
Targeting SLC36-transporter with amino acid derivative suppresses tumour growth. Dissected eye epithelial tissue stained with DAPI (red) from *ras1*^*G12V*^*;csk*^*−/−*^ animals raised on HSD (**a**), HSD supplemented with 10 mM L-tryptophan (trp) (**b**), HSD supplemented with 10 mM Indole-5-carboxylic acid (I5C) (**c**) and HSD supplemented with 10 mM Indole-3-propionic acid (IPA) (**d**). Scale bar, 250 μm. Dissected eye epithelial tissue stained with DAPI (red) from *ras1*^*G12V*^*;csk*^*−/−*^ animals raised on CD (**e**) and CD supplemented with 10 mM IPA (**f**). Scale bar, 250 μm. Third-instar larvae from *ras1*^*G12V*^*;csk*^*−/−*^ animals raised on HSD (**g**) and HSD supplemented with 10 mM IPA (**h**). Scale bar, 500 μm. **i** Pupariation percentage of *ras1*^*G12V*^*;csk*^*−/−*^ animals raised on HSD and HSD supplemented with 10 mM IPA. Results are shown as mean ± SEM. Data from total *n* = 120 (HSD) and *n* = 75 (HSD + IPA) from four independent experiments. Data were analysed by two-tailed unpaired Student’s *t* test. Asterisks indicate statistically significant difference (***P < 0.001). Dissected eye epithelial tissue stained with DAPI (red) from *ras1*^*G12V*^*;csk*^*−/−*^*,path* animals raised on CD supplemented with 100 mM L-proline (**j**), CD supplemented with 100 mM L-proline and 10 mM IPA (**k**) and CD supplemented with 250 mM L-proline and 10 mM IPA (**l**). Scale bar, 250 μm. **m** Working model illustrating dual-layered coordination of tumour metabolic response: (1) at the whole organism level—branchless (Bnl)-dependent induction of cachexia-like muscle wasting and systemic amino acid release (blue arrows)—and (2) at the tumour-autonomous level—Yorkie-dependent induction of amino acid transporter expression (red arrows). The tumour-autonomous SIK-Yki-Wg-InR circuit was identified previously^14^. IPA represents a therapeutic strategy to break the nutritional circuit between muscle wasting and tumour growth by targeting the SLC36-family transporter Path. SIK Salt-inducible kinase, Yki Yorkie, Wg wingless, InR insulin receptor, IPA indole-3-propionic acid.

Our work highlights SLC36-family transporters as a target for therapeutic intervention in tumours with proline vulnerability. A previous in vitro study using a human cancer cell line explored amino acid and amino acid derivative specificities of SLC36A1^32^. By selecting competitive, non-transported inhibitors of SLC36A1 from this study, we performed a whole-animal *Drosophila* screen to identify candidates that suppress tumour growth and display minimal whole-animal toxicity. Feeding *ras1*^*G12V*^;*csk*^*−/−*^ animals an HSD supplemented with 5-Hydroxy-L-tryptophan led to animal lethality at the early larval stages, indicative of whole-animal toxicity. Feeding *ras1*^*G12V*^;*csk*^*−/−*^ animals an HSD supplemented with either L-tryptophan or Indole-5-carboxylic acid led to successful larval development, but had minimal inhibitory effect on tumour growth (Fig. 6a–c and Supplementary Fig. 8a). However, feeding *ras1*^*G12V*^;*csk*^*−/−*^ animals an HSD supplemented with Indole-3-propionic acid (IPA) dramatically suppressed tumour growth in a dose-dependent manner (Fig. 6d and Supplementary Fig. 8a–f). Strikingly, 66.2% of IPA-fed *ras1*^*G12V*^;*csk*^*−/−*^ animals achieved pupariation in HSD, revealing significant tumour-suppressing efficacy coupled with minimal whole-animal toxicity (Fig. 6g–i and Supplementary Fig. 8g). IPA feeding had no effect on benign tumour growth in *ras1*^*G12V*^;*csk*^*−/−*^ animals raised on CD (Fig. 6e, f and Supplementary Fig. 8h) supporting the notion that SLC36 inhibition only limits the growth of tumours with proline vulnerability. Furthermore, IPA suppressed *ras1*^*G12V*^;*csk*^*−/−*^*,path*-tumour growth in proline-fed animals in CD, and increasing the dietary concentrations of proline partially out-competed IPA-inhibition (Fig. 6j–l and Supplementary Fig. 8i). These data indicate that IPA specifically targets the proline dependency of tumour growth.

A previous report identified exogenous proline dependent cell lines, including pancreatic cancer cell line Panc 04.03^3^. The cell culture media DMEM lacks proline and other non-essential amino acids (NEAAs), whereas RPMI includes all NEAAs. Panc.04.03 cells exhibited increased proliferation in RPMI compared to DMEM (Supplementary Fig. 8j). Supplementation of proline into DMEM promoted cell proliferation, indicating that these cell lines are responsive to exogenous proline (Supplementary Fig. 8j). Furthermore, addition of IPA in the culture medium attenuated cell proliferation in proline-supplemented DMEM (Supplementary Fig. 8l, m). Although IPA had a minor effect on cell proliferation in DMEM, IPA suppresses enhanced cell proliferation which is mediated by exogenous proline (Supplementary Fig. 8k, m).

## Discussion

Muscle wasting is observed in chronic muscle wasting diseases due to cancer (cachexia), ageing/senescence (sarcopenia), myopathies and other metabolic diseases, as well as in acute conditions due to burns and sepsis^33^. In this study, we demonstrate that the combination of diet-induced obesity and tumour growth induces systemic muscle wasting (Fig. 1), associated with functional locomotion defect (Supplementary Fig. 1o, p). This muscle phenotype was not due to the inability to properly form muscle during development; the muscles are wholly formed at the early stage of larval development, and waste progressively at the later larval stage, as the tumours develop (Supplementary Fig. 1k–m). In addition, we demonstrate that the muscle phenotype observed in our model is unrelated to a developmentally related degeneration process (Supplementary Fig. 2).

Our study reveals a systemic amino acid-utilising circuit whereby HSD-enhanced tumours induce muscle wasting as a systemic metabolic network to drive tumourigenesis. A consequence of muscle wasting in *ras1*^*G12V*^;*csk*^*−/−*^ animals raised on HSD was increased release of proline into the circulation (Fig. 3a). Plasma amino acid profiling of patients with sarcopenia—a condition of muscle wasting associated with aging—showed elevated plasma proline levels^34^, indicating that elevated free circulating proline is a common feature of muscle wasting. We identify a proline vulnerability of HSD-enhanced tumours; SLC36-family amino acid transporter Path is required for tumour growth and exogenous proline promotes tumourigenesis through Path (Figs. 4 and 5). We highlight two layers of coordination in tumour metabolic response: (1) at the whole organism level—by promoting muscle wasting and systemic amino acid availability, and (2) at the tumour-autonomous level—by altering amino acid transporter repertoire (Fig. 6m).

Path and CG1139 have different transport characteristics for proline, with Path having a lower transport capacity compared to CG1139^30^. Despite this, our labelled proline uptake experiments indicated uptake of extracellular proline in Ras/Src/Path-tumours but not in Ras/Src/CG1139-tumours (Fig. 5j), suggesting that CG1139 and Path may exhibit different activities in the context of an oncogenic background. Consistent with a previous report^30^, our data support the signalling role of Path through activation of the Tor-S6K pathway. Proline metabolism has been demonstrated to support cancer clonogenicity and metastasis^3,35^. Furthermore, proline promotes cancer cell survival under nutrient-limited or hypoxic microenvironments^4,36^. Our data extend these studies to reveal a tumour-promoting role of proline in response to systemic host metabolic changes.

IPA specifically targets the proline dependency of tumour growth in *Drosophila* (Fig. 6). Furthermore, we demonstrate a functional effect of IPA on human cells (Supplementary Fig. 8j–m). A recent study demonstrated that proline uptake in Ras-driven tumour cells is much higher in spheroids in three-dimensional cultures—which better mimic conditions in vivo—compared to monolayers in two-dimensional cultures^35^, suggestive of a potentially stronger effect in tumours that are dependent on proline for growth in vivo. Proline is a limiting amino acid for protein synthesis in kidney cancers^37^. Therefore, reducing proline uptake with SLC36-inhibitors—as exemplified here by use of IPA (Fig. 6)—may warrant consideration as a therapeutic strategy to break the nutritional circuit between systemic muscle wasting and tumour growth in proline vulnerable cancers.

## Methods

### Fly stocks

*UAS-lacZ* (BDSC: 8529), *ecd*^*1*^ (BDSC: 218), *UAS-bnl*^*RNAi/TRiP*^ (BDSC: 34572)*, UAS-ImpL2*^*RNAi/TRiP*^ (BDSC: 55855), *UAS-Pvf2*^*RNAi/TRiP*^ (BDSC: 61955), *UAS-bnl* (BDSC: 64231), *mhc-gal4* (BDSC: 55133), *UAS-btl.λ* (BDSC: 29045), *UAS-rl*^*SEM*^ (BDSC: 59006), and *UAS-rl*^*RNAi/TRiP*^ (BDSC: 31387), *UAS-rl*^*RNAi/TRiP*^ (BDSC: 31524), *UAS-path*^*RNAi/TRiP*^ (BDSC: 64029), *UAS-wts* (BDSC: 44258), *UAS-Tor*^*TED*^ (BDSC: 7013), and *UAS-S6K*^*STDETE*^ (BDSC: 6914) flies were obtained from the Bloomington *Drosophila* Stock Centre.

*UAS-bnl*^*RNAi/GD*^ (VDRC: 5730), *UAS-ImpL2*^*RNAi/GD*^ (VDRC: 30930)*, UAS-Pvf1*^*RNAi/GD*^ (VDRC: 6173), *UAS-Pvf1*^*RNAi/KK*^ (VDRC: 102699), *UAS-Pvf2*^*RNAi/KK*^ (VDRC: 102072), *UAS-btl*^*RNAi/GD*^ (VDRC: 950), *UAS-btl*^*RNAi/KK*^ (VDRC: 110277), *UAS-mnd*^*RNAi/GD*^ (VDRC: 42485), *UAS-slif*^*RNAi/GD*^ (VDRC: 45590), *UAS-JhI-21*^*RNAi/GD*^ (VDRC: 45193)*, UAS-CG8785*^*RNAi/GD*^ (VDRC: 4650)*, UAS-path*^*RNAi/KK*^ (VDRC: 100519), *UAS-CG1139*^*RNAi/GD*^ (VDRC: 8907) and *UAS-CG1139*^*RNAi/KK*^ (VDRC: 102363) flies were obtained from the Vienna *Drosophila* Resource Centre.

The following stocks were kindly provided to us: *FRT82B, csk*^*Q156Stop*^ by A. O’Reilly and M. Simon, *ey(3.5)-FLP1* and *UAS-ras1*^*G12V*^ by G. Halder, *FB-gal4* by R. Kühnlein, *UAS-pathA* by J. Parrish, *UAS-CG1139* by D. Goberdhan and *UAS-Yki.V5* by K. Irvine.

To create eyeless-driven GFP-labelled clones, the MARCM system was used. Flies with the genotype *ey(3.5)-FLP1; act* > *y*+ *>* *gal4,UAS-GFP; FRT82B*, *tub-gal80* were crossed to flies with the following genotypes:

(1) *UAS-LacZ*; *FRT82B*; (2) *UAS-ras1*^*G12V*^; *FRT82B, csk*^*Q156Stop*^*/TM6b*; (3) *UAS-ras1*^*G12V*^; *FRT82B, csk*^*Q156Stop*^*, UAS-bnl*^*RNAi/GD*^*/TM6b*; (4) *UAS-ras1*^*G12V*^; *FRT82B, csk*^*Q156Stop*^*, UAS-bnl*^*RNAi/TRiP*^*/TM6b*; (5) *UAS-ras1*^*G12V*^; *FRT82B, csk*^*Q156Stop*^*, UAS-ImpL2*^*RNAi/GD*^*/TM6b*; (6) *UAS-ras1*^*G12V*^*, UAS-ImpL2*^*RNAi/TRiP*^; *FRT82B, csk*^*Q156Stop*^*/TM6b*; (7) *UAS-ras1*^*G12V*^; *FRT82B, csk*^*Q156Stop*^*, UAS-Pvf1*^*RNAi/GD*^*/TM6b*; (8) *UAS-ras1*^*G12V*^*, UAS-Pvf1*^*RNAi/KK*^; *FRT82B, csk*^*Q156Stop*^*/TM6b*; (9) *UAS-ras1*^*G12V*^*, UAS-Pvf2*^*RNAi/KK*^; *FRT82B, csk*^*Q156Stop*^*/TM2*; (10) *UAS-ras1*^*G12V*^*, UAS-Pvf2*^*RNAi/TRiP*^; *FRT82B, csk*^*Q156Stop*^*/TM2*; (11) *UAS-ras1*^*G12V*^*, UAS-btl*^*RNAi/GD*^; *FRT82B, csk*^*Q156Stop*^*/TM6b*; (12) *UAS-ras1*^*G12V*^*, UAS-btl*^*RNAi/KK*^; *FRT82B, csk*^*Q156Stop*^*/TM6b*; (13) *UAS-ras1*^*G12V*^; *FRT82B, csk*^*Q156Stop*^*, UAS-mnd*^*RNAi/GD*^*/TM6b*; (14) *UAS-ras1*^*G12V*^; *FRT82B, csk*^*Q156Stop*^*, UAS-JhI-21*^*RNAi/GD*^*/TM6b*; (15) *UAS-ras1*^*G12V*^; *FRT82B, csk*^*Q156Stop*^*, UAS-slif*^*RNAi/GD*^*/TM6b*; (16) *UAS-ras1*^*G12V*^; *FRT82B, csk*^*Q156Stop*^*, UAS-CG8785*^*RNAi/GD*^*/TM6b*; (17) *UAS-ras1*^*G12V*^*, UAS-path*^*RNAi/KK*^; *FRT82B, csk*^*Q156Stop*^*/TM6b*; (18) *UAS-ras1*^*G12V*^*, UAS-path*^*RNAi/TRiP*^; *FRT82B, csk*^*Q156Stop*^*/TM6b*; (19) *UAS-ras1*^*G12V*^; *FRT82B, csk*^*Q156Stop*^*, UAS-CG1139*^*RNAi/GD*^*/TM6b*; (20) *UAS-ras1*^*G12V*^*, CG1139*^*RNAi/KK*^; *FRT82B, csk*^*Q156Stop*^*/MKRS*; (21) *UAS-ras1*^*G12V*^; *FRT82B, csk*^*Q156Stop*^*, UAS-wts*; (22) *UAS-ras1*^*G12V*^; *FRT82B, csk*^*Q156Stop*^*, UAS-yki*; (23) *UAS-ras1*^*G12V*^; *FRT82B, csk*^*Q156Stop*^*, UAS-path/TM6b*; (24) *UAS-ras1*^*G12V*^, *UAS-CG1139*; *FRT82B, csk*^*Q156Stop*^*/MKRS*; (25) *FRT82B, UAS-path*; (16) *UAS-CG1139; FRT82B*; (26) *UAS-ras1*^*G12V*^*, UAS-Tor*^*TED*^; *FRT82B, csk*^*Q156Stop*^*/MKRS*; (27) *UAS-ras1*^*G12V*^; *FRT82B, UAS-S6K*^*STDETE*^*, csk*^*Q156Stop*^*/TM6b*; (28) *UAS-ras1*^*G12V*^*, UAS-Tor*^*TED*^; *FRT82B, csk*^*Q156Stop*^*, UAS-path/TM6b*.

To block ecdysone signalling, we used a temperature-sensitive allele of ecdysoneless (*ecd*^*1*^). Flies with the genotype ey*(3.5)-FLP1; act* > *y* + > *gal4, UAS-GFP; ecd*^*1*^*, FRT82B*, *tub-gal80* were crossed with *UAS-ras1*^*G12V*^; *ecd*^*1*^*, FRT82B, csk*^*Q156Stop*^*/TM6b* flies and reared at 25 °C. At second instar larval stage (day 8 in 25 °C), cultures were switched to a restrictive temperature of 29 °C.

To carry out fat body-specific genetic alterations, *FB-gal4* flies were crossed with *UAS-LacZ* or *UAS-bnl*.

To carry out muscle-specific genetic alterations, *mhc-gal4* flies were crossed with: (1) *UAS-LacZ*, (2) *UAS-btl.λ*, (3) *UAS-rl*^*SEM*^, (4) *UAS-btl.λ, rl*^*RNAi/TRiP31387*^ and (5) *UAS-btl.λ, rl*^*RNAi/TRiP31524*^.

### Cultures

Cultures were carried out on a modified Bloomington semi-defined medium (https://bdsc.indiana.edu/information/recipes/germanfood.html), containing sucrose (0.15 and 1.0 M in CD and HSD, respectively) as the only purified sugar source. Ingredients were obtained from; Agar (Fisher Scientific; BP2641-1), Brewer’s Yeast (MP Biomedicals; 903312, Lot: BCBN0171V), Yeast Extract (Sigma-Aldrich; 70161), Peptone (Sigma-Aldrich; 82303), Sucrose (Fisher Scientific; S/8560/63), Magnesium sulfate hexahydrate (Fluka; 00627), Calcium chloride dihydrate (Sigma-Aldrich; 223506), Propionic acid (Sigma-Aldrich; P1386), p-Hydroxy-benzoic acid methyl ester (Sigma-Aldrich; H5501). For dietary supplementation experiments, L-proline (Sigma-Aldrich; P5607), D-proline (Sigma-Aldrich; 858919), L-glycine (VWR Chemicals; 101196X), L-alanine (Sigma-Aldrich; 7627), L-tryptophan (Sigma-Aldrich; T0254), Indole-5-carboxylic acid (Sigma-Aldrich; I5400) and IPA (Sigma-Aldrich; 57400) were added to CD or HSD. Cultures were performed at 25 °C unless otherwise noted. HSD feeding led to a developmental delay in reaching the third-instar larval stage; all experiments were performed on late third-instar larvae at day 8 after egg laying (CD) and day 14 after egg laying (HSD) unless otherwise noted.

### Cell cultures and proliferation assay

Pancreatic cancer cell line Panc 04.03 was obtained from the American Type Culture Collection. Cells were plated in 96-well plates in a density of 2500 cells/well and cultured in RPMI, DMEM, DMEM + 1 mM proline, with or without 1 mM IPA. All media was supplemented with 2 mM glutamine, 100 U/mL penicillin/streptomycin and 10% dialysed foetal calf serum (3000 Da MWCO) in a humidified incubator at 37 °C, 5% CO_2_. Proliferation was measured for 120 h using the IncuCYTE live cell imaging system (Essen Biosciences). Datapoints were taken every 2 h. Growth rates were calculated using the Growthcurver package in R (ver. 0.3.0).

### Immunohistochemistry

Tissue was dissected in phosphate-buffered saline (PBS), fixed in 4% PFA/PBS for 30′ on ice, washed in PBS-T, incubated with primary antibody in PAXDG (PBS containing 1% BSA, 0.3% Triton X-100, 0.3% deoxycholate and 5% goat serum), followed by washing and incubation with secondary antibody in PAXDG and subsequent mounting in Vectashield with DAPI (Vector Laboratories). Primary antibodies used were: rat anti-myosin (ab51098; Abcam; 1:100) and mouse anti-pERK (M-8159; Sigma; 1:50). Polyclonal rabbit anti-CG1139 (1:200) was purified by New England Peptide Inc., MA, USA. An amino acid sequence 26–40 was selected as the epitope. The following primary antibodies were kindly gifted to us: rat anti-bnl (M. Krasnow; 1:50), guinea pig anti-Path (J. Parrish; 1:200) and rabbit anti-phospho-Drosophila S6 (pS6) (A. Teleman; 1:500). Secondary antibodies used were: Alexa Fluor 488 and 568 conjugated anti-rat antibody (A-11006 and A-11077; Thermo Fisher Scientific; 1:200), Alexa Fluor 568 conjugated anti-mouse antibody (A-11031; Thermo Fisher Scientific; 1:200), Alexa Fluor 568 conjugated anti-rabbit antibody (A-11036; Thermo Fisher Scientific; 1:200) and Alexa-568 conjugated anti-guinea pig antibody (A-11075; Thermo Fisher Scientific, 1:200). Rhodamine phalloidin (R415; Invitrogen; 1:500) was used to visualise F-actin.

### Nile red staining

Tissue was dissected in PBS, fixed in 4% PFA/PBS for 30′ on ice, incubated with 10 μg/mL Nile Red stain (N1142; Thermo Fisher Scientific) in PBS for 30′ on ice followed by washing and subsequent mounting in Vectashield with DAPI.

### Acridine orange staining

Midguts were dissected in PBS on ice, incubated in 5 μg/ml of acridine orange (A1301: Thermo Fisher Scientific), followed by washing and subsequent mounting in Vectashield with DAPI. Samples were imaged immediately with the Leica SP5 II confocal microscope and Leica Application Suite Advanced Fluorescence software (Leica Microsystems).

### Blue dye feeding assay

Larvae were placed on food containing 1% FCF-blue dye for 90 min and washed in dH_2_O to remove excess dye from their cuticle. Larvae were imaged with Leica DFC 3000 G digital camera to visualise blue food in the gut, or retained for feeding quantification. For feeding quantification five larvae were pooled into 1.5 ml Eppendorf tubes each containing 70 μl dH2O, followed by homogenisation with motorised pestle and mortar, and centrifuged for 5 min at 10,000 RCF. 40 μl of supernatant from each sample was transferred to a transparent 96-well plate and absorbance was measured at 629 nm in BMG Labtech FLUOstar Omega Microplate reader.

### Smurf gut integrity assay

Larvae were placed on food containing 1% FCF-blue dye overnight, with a minimum of ten animals per vial. Larvae were washed in dH_2_O to remove excess dye from their cuticle and scored as ‘Smurfs’ if blue dye was only visible in the gut, or ‘Non-smurfs’, if blue dye was visible across the whole animal.

### Muscle wasting quantification

Individual larvae were washed and dissected in PBS. Larval body-wall muscle was stained with Rhodamine phalloidin (R415; Invitrogen; 1:500) to visualise F-actin. Individual animals were scored into categories each representing the strength of muscle wasting from either ‘none’; ‘minor’; ‘moderate’ or ‘strong’ (Supplementary Fig. 1f–i). Scores were assigned based on wasting of the ventral longitudinal, lateral oblique and lateral longitudinal muscles centred around the fourth abdominal segment (A4) of the larval body wall and each hemisegment was used as an internal control (Supplementary Fig. 1e). A minimum of 30 animals were scored per condition. Body-wall muscle staining and quantification was performed on animals from day 8 (CD), day 10 (early stage; HSD), day 14 (mid stage; HSD) and day 16 (late stage; HSD) after egg laying.

### Microscopy and imaging

Larval images were acquired with Leica M165 FC fluorescent microscope equipped with Leica DFC 3000 G digital camera. Eye disc images were acquired with Leica M165 FC fluorescent microscope equipped with S-View SXY-I30 digital camera. Confocal images were acquired with Leica SP5 II confocal microscope and Leica Application Suite Advanced Fluorescence software (ver. 4.6.1) (Leica Microsystems). Larval GFP/brightfield overlay images were compiled with Adobe Photoshop software.

### Immunostaining quantification

Quantification of staining intensity for Bnl, Path, CG1139 and phospho-Drosophila ribosomal protein S6 (pS6), each stained with Alexa-568 secondary antibody, was carried out on GFP-positive clones. A minimum of three samples per condition were analysed in ImageJ software (ver. 2.0.0-rc-43) using a custom-made macro written by D. Dormann. RGB images were first converted to tagged image file format (tiff), channels were split and the green channel was thresholded for GFP intensity to allow for the identification of GFP-positive clone area. For the GFP-positive clone area, mean pixel intensity was calculated across the red channel.

### Eye disc area quantification

A minimum of three samples per condition were analysed in ImageJ software (ver. 2.0.0-rc-43) using a custom-made macro written by D. Dormann. Images were first converted to tiff format and RGB channels were split. The eye disc area was manually defined, based on the outline of DAPI staining in the red channel. The green channel was thresholded for GFP intensity to allow for the identification of GFP-positive clones within the user-defined eye disc. Clone area was subsequently calculated.

### Larval video tracking and analysis

Larvae were maintained in normal culture conditions up until the point of video recording. Individual larvae were picked and placed onto the centre of an agar plate at room temperature and recorded immediately. Video plate recordings were carried out with use of a Teledyne DALSA Genie Nano Camera (G3-GM11-M2420) using Gecko GigE Video Recorder software (v2.0.3.1; www.visionexperts.co.uk), at 25 frames per second, ensuring a sharp contrast between the larva and background. Twenty individual larvae were recorded per condition up to a maximum of 5 min or until the larva was no longer in the field of view. Individual videos—each corresponding to one larva—were segmented, tracked and skeletonised using Tierpsy Tracker^16^. Two hundred and fifty-six defined features, previously determined to be useful in classifying behaviour in *C. elegans*, were extracted from the tracking data and compared between conditions^17^. Correction for multiple testing was applied using the Benjamini–Hochberg procedure to control the false discovery rate to 0.05^38^.

### Quantitative RT-PCR

Larvae were washed in PBS and dissected in RNALater solution. Total RNA was extracted from pooled dissected tissue using the QIAGEN RNeasy Mini Kit. First-strand DNA was synthesised with the iScript cDNA synthesis kit (Bio-Rad). PCR was performed by mixing cDNA samples with iTaq Universal SYBR Green Supermix (Bio-Rad), ROX passive reference dye (Bio-Rad) and the relevant primers in a 96-well plate. Analysis was carried out on a 7900HT Real-Time PCR system with Applied Biosystems Software (SDS v2.4) (Applied Biosystems). Data analysis was carried out in Microsoft Excel (16.16.4). At least three independent biological replicates and two technical replicates were used. Expression values were normalised to *Act88F*. Primers used are listed in Supplementary Table 1.

### RNA-sequencing

RNA from pooled dissected tissue was extracted with the QIAGEN RNeasy Mini Kit. A minimum of three samples were prepared for each genotype. RNA-seq libraries were prepared from a minimum of 10 ng of total RNA using the Illumina Truseq mRNA stranded library prep kit (Illumina, San Diego, USA) according to the manufacturer’s protocol. Library quality was checked on a Bioanalyser HS DNA chip and concentrations were estimated by Qubit measurement. Libraries were pooled in equimolar quantities and sequenced on a Hiseq2500 using paired end 100 bp reads. At least 35 million reads passing filter were achieved per sample. After demultiplexing, raw or trimmed RNA-Seq reads were aligned against Ensembl *Drosophila* genome reference sequence assembly (dm3) and transcript annotations using TopHat2^39^. Trim Galore, developed at The Babraham Institute by Felix Krueger (http://www.bioinformatics.babraham.ac.uk/projects/trim_galore/), was applied for trimming adaptor and low-quality reads. For differential gene expression analysis, gene-based read counts were then obtained using the featureCounts function from Rsubread Bioconductor package^40^. Differential expression analysis was performed on the counts data using DESeq2 Bioconductor package^41^. From tumour RNA-sequencing data, putative tumour-secreted factors were annotated based on identification of genes labelled with the GO term ‘extracellular’ or presence of the signal peptide sequence as identified by the signal peptide database (http://www.signalpeptide.de).

### Gene set enrichment analysis

GSEA was carried out using a ranked gene list based on Wald statistics from DESeq2 results^42^. MSigDB gene sets from ‘H’, ‘C2’ and ‘C5’ collections were used and only considered where FDR *q* value < 0.25. Normalised enrichment scores were calculated for multiple terms within functional categories. Results were displayed as cumulative normalised enrichment score, as previously published^43^.

### Haemolymph sample preparation

Pooled haemolymph was collected from multiple animals on ice to a combined minimum sample volume of 25 μl. Each sample was mixed with 225 μl of methanol containing internal standards (IS) (50 μm). Then, chloroform (250 μl) and Milli-Q water (100 μl) were added, mixed and centrifuged. The water layer was filtrated through a 5-kDa cut-off filter to remove macromolecules. Filtrate was centrifugally concentrated and resuspended in 25 μl of ultrapure water prior to measurement.

### Haemolymph metabolomic analyses by capillary electrophoresis mass spectrometry (CE-MS)

Targeted metabolomic analyses were performed by the Human Metabolome Technologies Inc., Yamagata, Japan (HMT). CE-MS was performed using Agilent CE-TOFMS Machine (CE-TOFMS) and Agilent 6460 TripleQuad LC/MS Machine (CE-QqQMS)(Agilent Technologies). Peaks detected in CE-TOFMS analysis were extracted using automatic integration software (MasterHands ver.2.17.1.11 developed at Keio University)^44^ and those in CE-QqQMS analysis were extracted using automatic integration software (MassHunter Quantitative Analysis B.06.00 Agilent Technologies, Santa Clara, CA, USA) in order to obtain peak information including *m/z*, migration time (MT) and peak area. Putative metabolites were assigned from peak alignments based on the HMT metabolite database on the basis of *m/z* and MT. Relative peak values were calculated based on IS and normalised to sample volumes. Absolute quantification was calculated by normalising the peak area of each metabolite with respect to the area of the IS and by using standard curves, which were obtained by three-point calibrations. For haemolymph samples, 214 metabolites (135 metabolites in cation mode and 79 metabolites in anion mode, respectively) were annotated based on the HMT metabolite database, including 3-methylhistidine and proline.

### Sample preparation for targeted proline quantification

Frozen tissue samples (15 pooled tumours per replicate) or haemolymph samples (1 µL per replicate) were extracted with 300 µL of extraction solvent (water/methanol, 20:80 v/v). The extraction solvent contained IS at 150 ng/mL using either L-proline-2,5,5-d3 (Sigma) or ^13^C_5_^15^N-L-proline (Sigma), depending on the experiment. Samples were sonicated using an ultrasonic water bath (15 min) and centrifuged (13,000 × *g*, 10 min, 5 °C). An aliquot of the supernatant (180 µL) was then filtered into a 96-well V-bottom plate using a 96-well filter plate (PTFE 0.45 µm). Pooled quality controls were created for each batch by pooling equal aliquots of each study sample in the batch, in order to assess technical reproducibility.

### Targeted proline quantification by LC-MS/MS

Chromatographic analyses were carried out on a Vanquish Flex Binary UHPLC system (Thermo Scientific Inc., MA, USA) coupled to a benchtop hybrid quadrupole-Orbitrap Q Exactive mass spectrometer (Thermo Scientific Inc., Bremen, Germany). Separation was achieved using an Accucore 150 Amide HILIC column (Thermo Scientific, 150 × 2.1 mm, 2.6 µm) equipped with a guard column (Thermo Scientific, 30 × 2.1 mm, 2.6 µm), both held at a temperature of 40 °C and a flow rate of 0.2 mL/min. Mobile phases were 10 mM aqueous ammonium acetate/acetonitrile (10:90 v/v) (solvent A) and 10 mM aqueous ammonium acetate/acetonitrile/water (10:10:80 v/v) (solvent B). The gradient elution was performed with a 25–47% solvent B gradient over 14 min, held at 47% B for 4 min and returning to 25% B at 20 min. The column was equilibrated for 10 min, yielding a total run time of 30 min. Ionisation was performed in the positive ionisation mode using a heated electrospray ionisation source, with the following parameters: spray voltage 3.0 KV, heater temperature 330 °C, capillary temperature 320 °C, S-lens RF level 50, sheath and auxiliary gas flow rate, 35 and 10 units, respectively. The mass accuracy was calibrated prior to sample analysis. Mass spectrometric data were acquired in profile mode using the parallel reaction monitoring method with compound-specific settings defined in the inclusion list (Supplementary Table 2). Further acquisition settings were MS2 resolution of 17,500 (at *m/z* 200) and an isolation window of *m/z* 1.2. Nitrogen was utilised as collision gas in the higher energy collisional dissociation cell with normalised collision energy as summarised in Supplementary Table 2. Automatic gain control was set to 1e5 and maximum injection time 100 ms. Xcalibur version 4.1 was used for data acquisition and processing. Specific fragment ions were used in the data processing in order to detect and quantify each compound unequivocally. Relative quantification is reported based on the peak area ratio of the analyte and the respective IS.

### Statistics and reproducibility

Unless otherwise stated, all statistical analyses were carried out with an unpaired, two-tailed Student’s *t* test in GraphPad Prism software (v.7.0d) where *P* ≤ 0.0001 = ****; 0.0001 < *P* ≤ 0.001 = ***; 0.001 < *P* ≤ 0.01 = **; 0.01 < *P* ≤ 0.05 = *; *P* > 0.05 = not significant. All experiments were independently repeated at least three times.

### Reporting summary

Further information on research design is available in the Nature Research Reporting Summary linked to this article.

## Supplementary information

Supplementary Information

Reporting Summary

## Data Availability

All raw RNA-sequencing data that support the findings of this study have been deposited in the GEO repository with the accession codes GSE139958. Other data that support the study are available from the corresponding author upon reasonable request.
