# Supplementary material for: Systemic muscle wasting and coordinated tumour response drive tumourigenesis
Source: Nat Commun. 2020 Sep 16;11:4653. doi: 10.1038/s41467-020-18502-9 (PMC7495438; doi:10.1038/s41467-020-18502-9)
Supplement: Supplementary file 1 — Supplementary Information [file 41467_2020_18502_MOESM1_ESM.pdf]

Supplementary Information

Systemic muscle wasting and coordinated tumour response drive tumourigenesis

Newton et al.

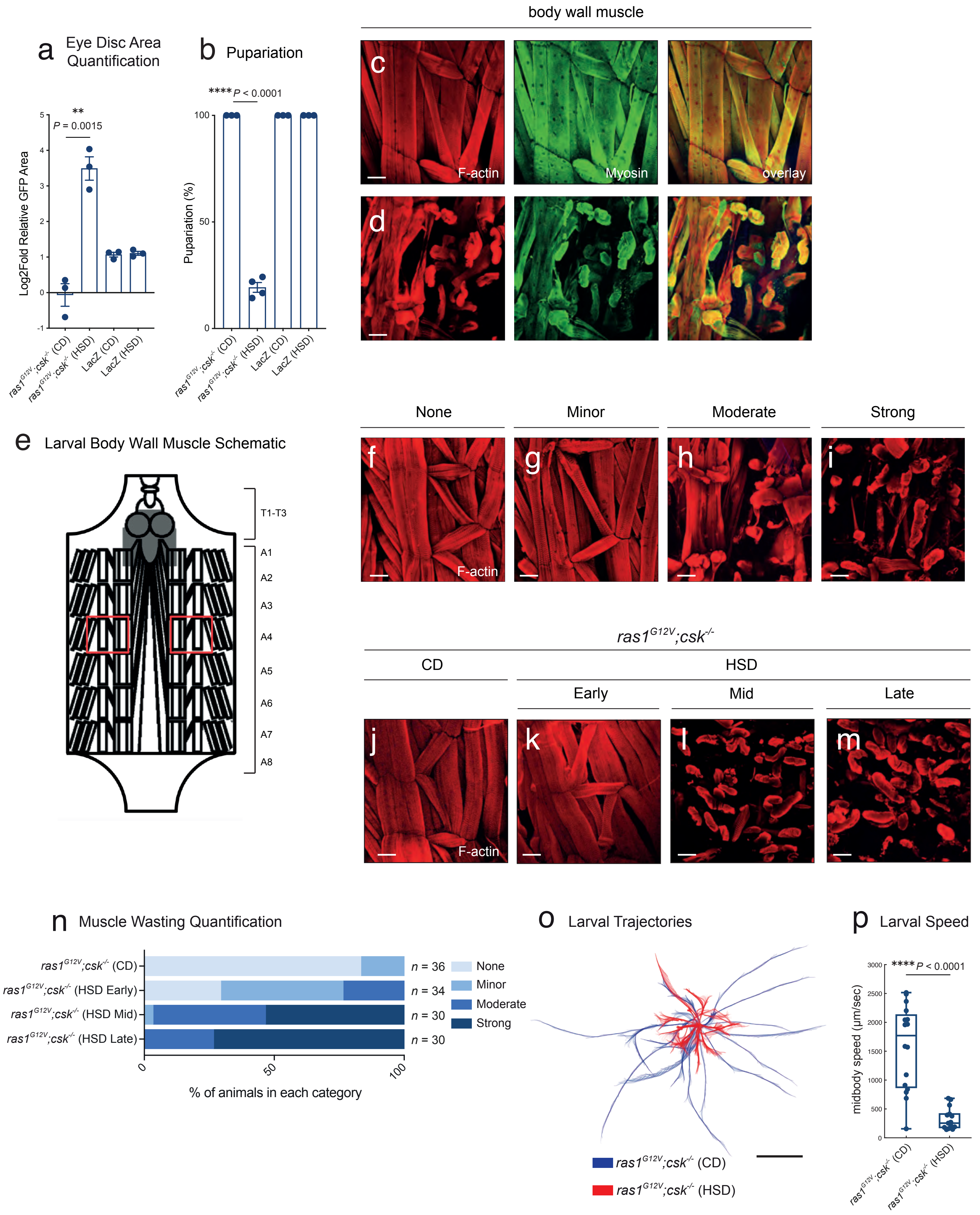

Supplementary Fig. 1

Supplementary Fig. 1. HSD-fed Ras/Src-animals exhibit progressive cachexia-like muscle wasting.

a, Eye disc area quantification of *ras1*^{G12V}*;csk*$^{-/-}$ and *LacZ* animals raised on CD or HSD. Results are shown as mean ± SEM. Data from n = 3 biologically independent samples. Data were analysed by two-tailed unpaired Student's *t*-test. Asterisks indicate statistically significant difference (**$P < 0.01$). **b**, Pupariation percentage of *ras1*^{G12V}*;csk*$^{-/-}$ and *LacZ* animals raised on CD or HSD. Results are shown as mean ± SEM. Data from total n = 76 (*ras1*^{G12V}*;csk*$^{-/-}$ CD), n = 120 (*ras1*^{G12V}*;csk*$^{-/-}$ HSD), n = 73 (*LacZ* CD), and n = 70 (*LacZ* HSD) from 4 independent experiments. Data were analysed by two-tailed unpaired Student's *t*-test. Asterisks indicate statistically significant difference (****$P < 0.0001$). **c**, **d**, F-actin (red) and anti-myosin (green) staining of dissected larval body wall muscle tissue from *ras1*^{G12V}*;csk*$^{-/-}$ third-instar larvae raised on CD (**c**) or HSD (**d**). Scale bar, 100 μm. **e**, Schematic of larval body wall muscle upon dissection from anterior (top) to posterior (bottom). The larval body wall contains 3 thoracic segments (T1-T3) and 9 abdominal segments (A1-A9). Abdominal muscles from segments A1-A8 are labelled. Red boxes outline regions of the body wall muscle which are imaged, containing the ventral longitudinal, lateral oblique and lateral longitudinal muscles centred around the fourth abdominal segment (A4). **f-i**, Representative images of dissected larval body wall muscle stained with phalloidin to visualise F-actin. Muscle wasting scores increase in strength from left to right: "None" (**f**), "Minor" (**g**), "Moderate" (**h**) and "Strong" (**i**). Scale bar, 100 μm. **j-m**, Dissected larval body wall muscle of *ras1*^{G12V}*;csk*$^{-/-}$ animals raised on CD (**j**) or on HSD at Early (**k**), Mid (**l**) and Late stage (**m**) Scale bar, 100 μm. **n**, Matching body wall muscle wasting quantification of *ras1*^{G12V}*;csk*$^{-/-}$ animals raised on CD and HSD. **o**, Individual larval trajectories from video tracking analysis of *ras1*^{G12V}*;csk*$^{-/-}$ third-instar larvae raised on CD (red; n =16), or HSD (blue; n = 14). Scale bar, 1 cm. **p,** Average midbody speed of *ras1*^{G12V}*;csk*$^{-/-}$ third-instar larvae raised on CD (n =16), or HSD (n = 14) with results shown in boxplot format with each data point representing one individual animal. Box plots: line, median; box, 75th–25th percentiles; whiskers, minimum to maximum. Data from n = 16 (*ras1*^{G12V}*;csk*$^{-/-}$ CD), and n = 14 (*ras1*^{G12V}*;csk*$^{-/-}$ HSD) animals. Data were analysed by two-tailed unpaired Student's *t*-test. Asterisks indicate statistically significant difference ($P < 0.0001$).

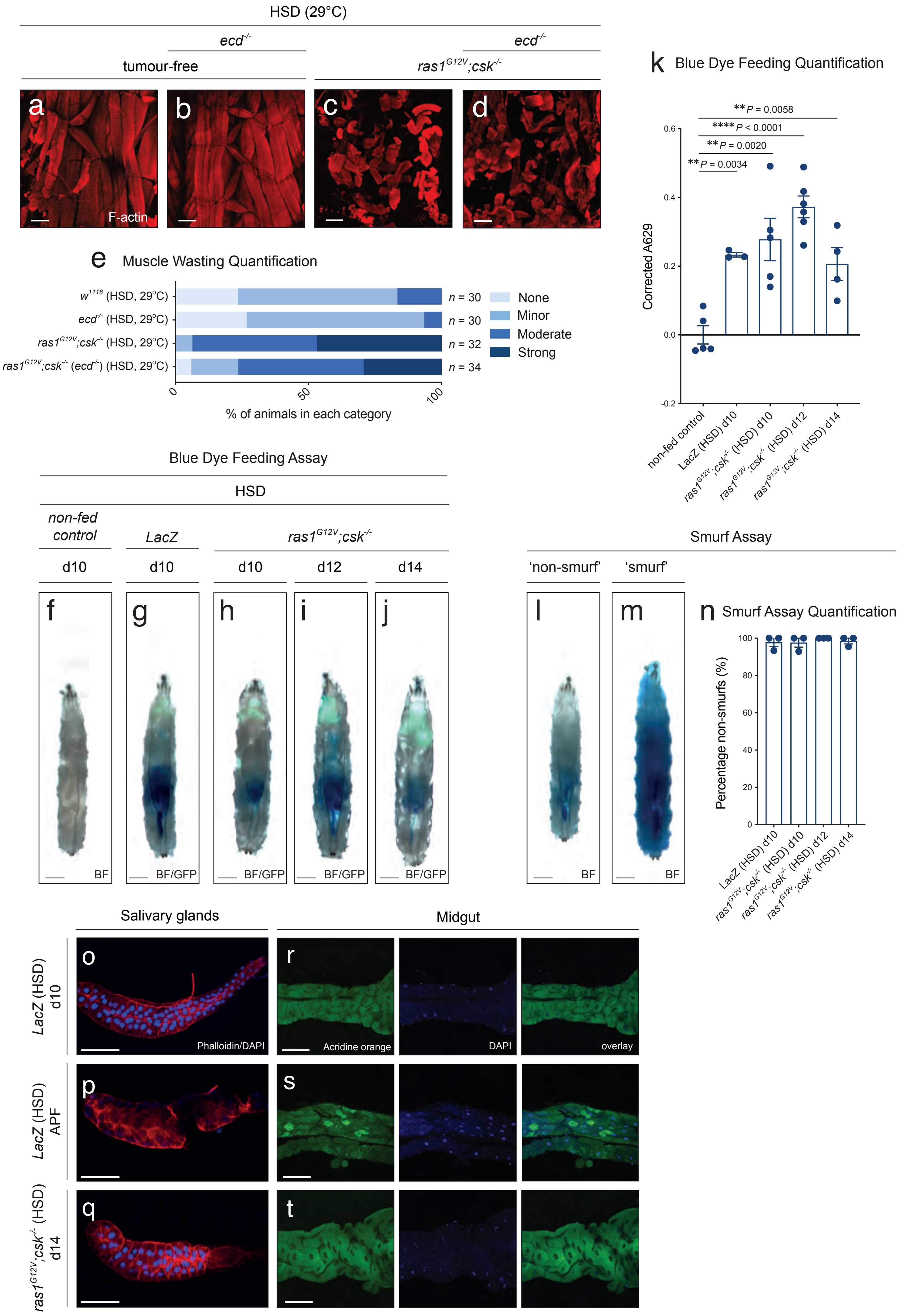

Supplementary Fig. 2

Supplementary Fig. 2. Muscle wasting occurs independent of ecdysone-mediated histolysis. **a-d**, F-actin (red) staining of dissected larval body wall muscle tissue from tumour free (**a, b**) and *ras1*^{G12V}*;csk*$^{-/-}$ (**c, d**) animals in *ecd*$^{-/-}$ third-instar larvae (**b, d**) raised on HSD for 8 days in 25°C plus 6 days after temperature shift to 29°C. Scale bar, 100 µm. **e**, Matching body wall muscle wasting quantification. **f-j**, Blue-dye feeding of larvae in non-fed control animals at day 10 after egg laying (AEL) (**f**), *LacZ* animals at day 10 AEL (d10) (**g**), *ras1*^{G12V}*;csk*$^{-/-}$ animals at day 10 AEL (d10) (**h**), *ras1*^{G12V}*;csk*$^{-/-}$ animals at day 12 AEL (d12) (**i**), and *ras1*^{G12V}*;csk*$^{-/-}$ animals at day 14 AEL (d14) (**j**). Scale bar, 500 µm. **k**, Quantification of blue dye feding assay. Results are shown as mean ± SEM. Data from n = 6 (non-fed control), n = 4 (*LacZ* HSD d10), n = 5 (*ras1*^{G12V}*;csk*$^{-/-}$ HSD d10), n = 6 (*ras1*^{G12V}*;csk*$^{-/-}$ HSD d12), and n = 4 (*ras1*^{G12V}*;csk*$^{-/-}$ HSD d14) biologically independent samples. Data were analysed by two-tailed unpaired Student's *t*-test. Asterisks indicate statistically significant difference (**P < 0.01; ****P < 0.0001). **l**, **m**, Representative images of larvae subjected to Smurf gut permeability assay. Scale bar, 500 µm. **n**, Quantification of Smurf assay. Results are shown as mean ± SEM. Data from total n = 36 (*LacZ* HSD d10), n = 45 (*ras1*^{G12V}*;csk*$^{-/-}$ HSD d10), n = 37 (*ras1*^{G12V}*;csk*$^{-/-}$ HSD d12), and n = 47 (*ras1*^{G12V}*;csk*$^{-/-}$ HSD d14) from 3 independent experiments. **o-q**, F-actin (red) staining with DAPI (blue) of salivary glands from *LacZ* animals at day 10 AEL (d10) (**o**), *LacZ* animals after pupariam formation (APF) (**p**), and *ras1*^{G12V}*;csk*$^{-/-}$ animals at day 14 AEL (d14) (**q**) raised on HSD. Scale bar, 100 µm. **r-t,** Acridine orange staining (green) with DAPI (blue) of midgut from *LacZ* animals at day 10 AEL (d10) (**r**), *LacZ* animals at pupariam formation (APF) (**s**), and *ras1*^{G12V}*;csk*$^{-/-}$ animals at day 14 AEL (d14) (**t**) raised on HSD. Scale bar, 100 µm.

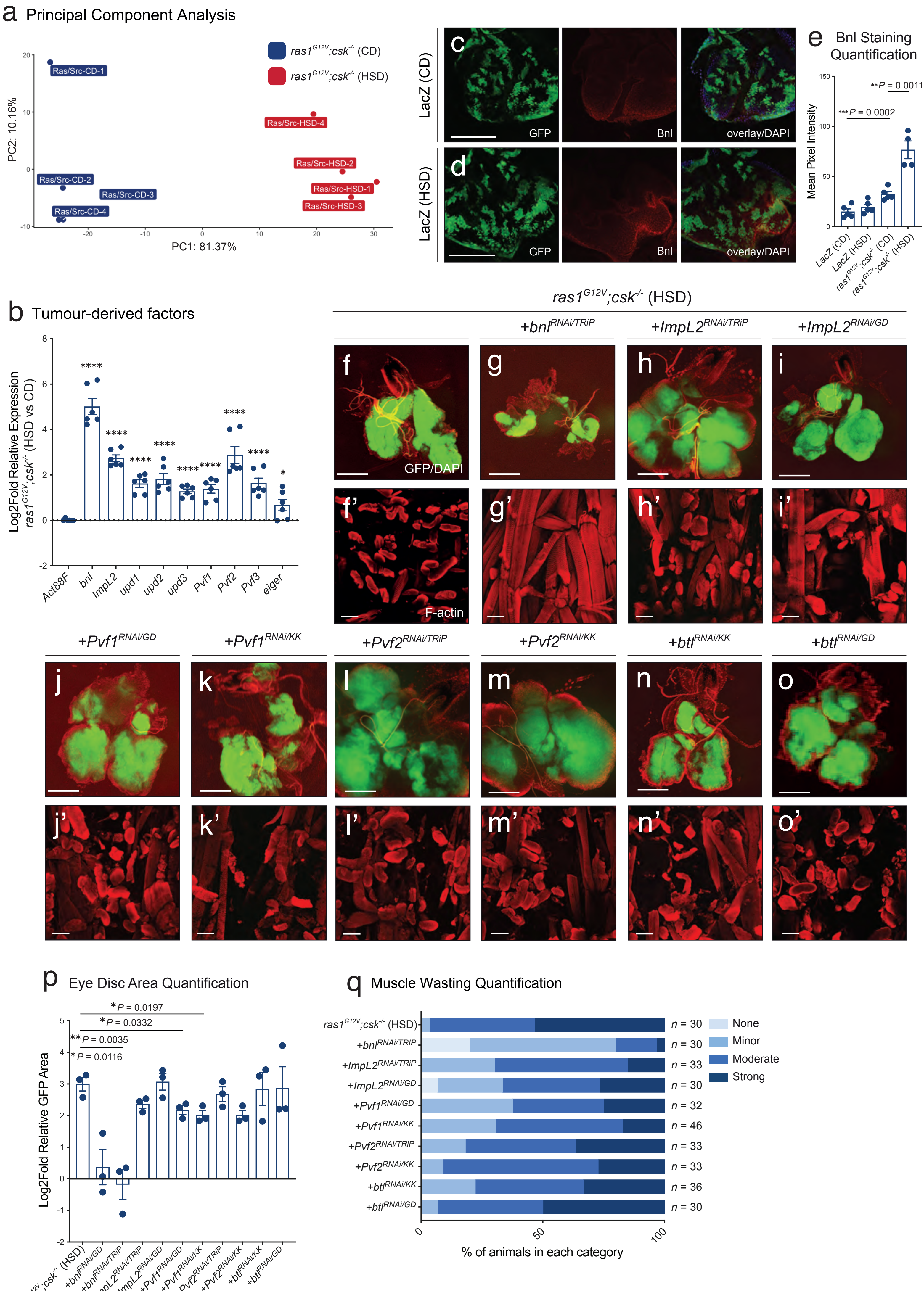

Supplementary Fig. 3

Supplementary Fig. 3. Identification of branchless as a tumour-derived factor that contributes to muscle wasting and tumour growth.

a, Principal Component Analysis of RNA-sequencing data from dissected tumour tissue in *ras1*^{G12V}*;csk*$^{-/-}$ animals raised on CD (blue) or HSD (red). *n* = 4 per condition. **b,** qPCR validation of candidate tumour-derived factors—*bnl*, *ImpL2*, *upd1*, *upd2*, *upd3*, *Pvf1*, *Pvf2*, *Pvf3* and *eiger*—initially identified by RNA-sequencing. Gene expression levels are shown as log2-relative fold change between *ras1*^{G12V}*;csk*$^{-/-}$ animals raised on HSD compared to animals raised on CD, as determined by qPCR. Samples are normalised to *Act88F*. Results are shown as mean ± SEM. Data from *n* = 6 biologically independent samples. Data were analysed by two-tailed unpaired Student's *t*-test. Asterisks indicate statistically significant difference (**P* = 0.0275; *****P* < 0.0001). **c, d,** Anti-Bnl staining (red) of dissected eye tissue from *LacZ* animals raised on CD (**c**) or HSD (**d**). Scale bar, 40 µm. **e**, Matching Bnl staining quantification. Results are shown as mean ± SEM. Data from *n* = 5 (*LacZ* CD), *n* = 5 (*LacZ* HSD), *n* = 5 (*ras1*^{G12V}*;csk*$^{-/-}$ CD), and *n* = 4 (*ras1*^{G12V}*;csk*$^{-/-}$ HSD) biologically independent samples. Data were analysed by two-tailed unpaired Student's *t*-test. Asterisks indicate statistically significant difference (***P* < 0.01; ****P* < 0.001). **f-o,** Dissected eye epithelial tissue stained with DAPI (red) from *ras1*^{G12V}*;csk*$^{-/-}$ (**f**), *ras1*^{G12V}*;csk*$^{-/-}$*,bnl*$^{RNAi/TRiP}$ (**g**), *ras1*^{G12V}*;csk*$^{-/-}$*,ImpL2*$^{RNAi/TRiP}$ (**h**), *ras1*^{G12V}*;csk*$^{-/-}$*,ImpL2*$^{RNAi/GD}$ (**i**), *ras1*^{G12V}*;csk*$^{-/-}$*,Pvf1*$^{RNAi/GD}$ (**j**), *ras1*^{G12V}*;csk*$^{-/-}$*,Pvf1*$^{RNAi/KK}$ (**k**), *ras1*^{G12V}*;csk*$^{-/-}$*,Pvf2*$^{RNAi/TRiP}$ (**l**), *ras1*^{G12V}*;csk*$^{-/-}$*,Pvf2*$^{RNAi/KK}$ (**m**), *ras1*^{G12V}*;csk*$^{-/-}$*,btl*$^{RNAi/KK}$ (**n**), and *ras1*^{G12V}*;csk*$^{-/-}$*,btl*$^{RNAi/GD}$ (**o**) third-instar larvae raised on HSD. Scale bar, 250 µm. **f'-o'**, Matching dissected larval body wall muscle stained with F-actin (red). Scale bar, 100 µm. **p,** Eye disc area quantification. Results are shown as mean ± SEM. Data from *n* = 3 biologically independent samples. Data were analysed by two-tailed unpaired Student's *t*-test. Asterisks indicate statistically significant difference (**P* < 0.05; ***P* < 0.01). **q,** Matching body wall muscle wasting quantification.

a Circulating Amino Acid Profile

Amino Acid	Fold Change (HSD v CD)	
	LacZ	Ras/Src
Ala	1.052	1.238
Arg	1.189	1.211
Asn	1.209	0.867
Asp	1.600	1.974
Cys	0.641	N/a
Gln	1.278	1.140
Glu	1.901	1.220
Gly	0.939	0.832
His	1.166	1.838
Ile	1.218	1.352
Leu	1.232	1.352
Lys	0.658	0.950
Met	0.665	0.660
Phe	0.990	0.849
Pro	1.659	2.262
Ser	1.234	1.577
Thr	0.800	0.677
Trp	0.862	0.908
Tyr	0.901	0.815
Val	0.913	1.189

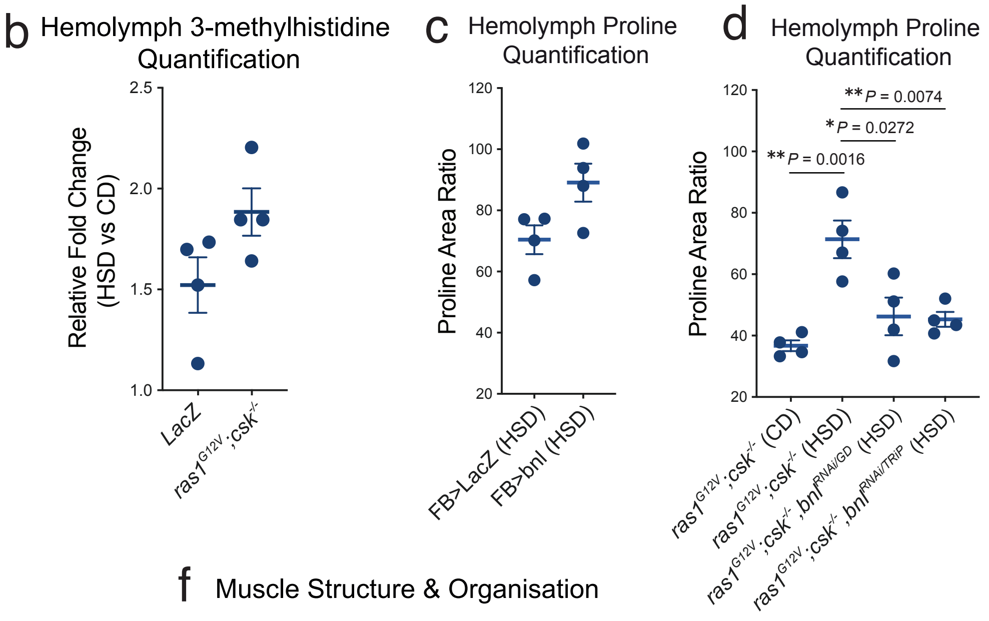

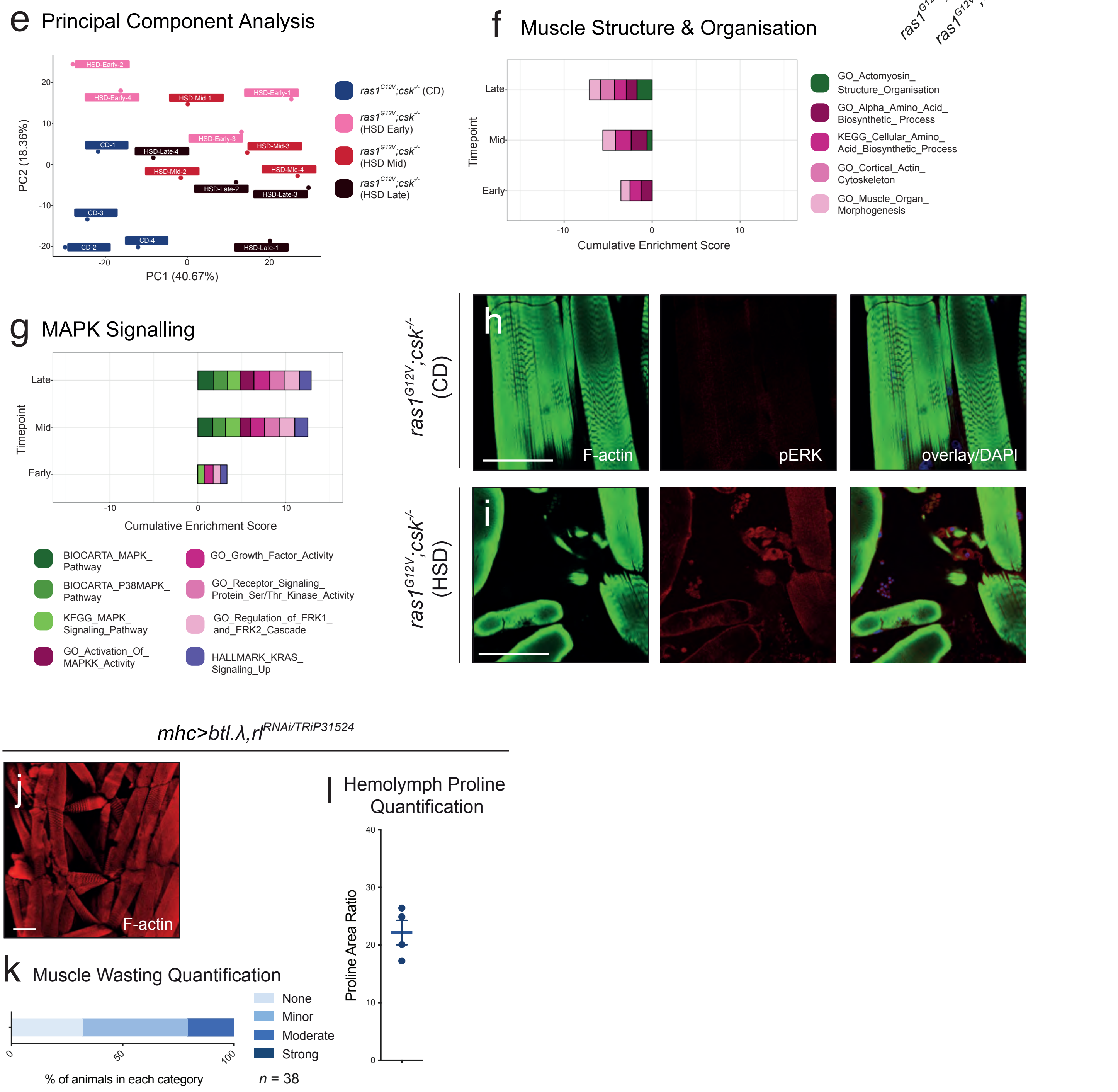

Supplementary Fig. 4

Supplementary Fig. 4. HSD-fed Ras/Src-animals promote muscle wasting via ERK-signalling.

a, Fold-change in hemolymph amino acid concentrations in *LacZ* and $ras1^{G12V};csk^{-/-}$ animals raised on HSD compared to CD. **b**, Circulating 3-methylhistidine levels depicted by relative peak area ratio from hemolymph in $ras1^{G12V};csk^{-/-}$animals raised on CD or HSD. Results are shown as mean ± SEM. Data from *n* = 4 biologically independent samples. **c**, Circulating proline levels depicted by relative peak area ratio from hemolymph in *FB>LacZ and FB>bnl* animals raised on HSD. Results are shown as mean ± SEM. Data from *n* = 4 biologically independent samples. **d**, Circulating proline levels depicted by relative peak area from hemolymph in $ras1^{G12V};csk^{-/-}$ animals raised on CD, $ras1^{G12V};csk^{-/-}$ animals raised on HSD, $ras1^{G12V};csk^{-/-},bnl^{RNAi/GD}$ animals raised on HSD and $ras1^{G12V};csk^{-/-},bnl^{RNAi/TRiP}$ animals raised on HSD. Results are shown as mean ± SEM. Data from *n* = 4 biologically independent samples. Data were analysed by two-tailed unpaired Student's *t*-test. Asterisks indicate statistically significant difference (**P* < 0.05; ***P* < 0.01). **e,** Principal Component Analysis of dissected muscle RNA-sequencing data from $ras1^{G12V};csk^{-/-}$ animals raised on CD (blue) and $ras1^{G12V};csk^{-/-}$ animals raised on HSD at Early (pink), Mid (red) and Late Stage (brown). *n* = 4 per condition. **f**, Gene Set Enrichment Analysis (GSEA) for the functional category 'Muscle Structure & Organisation' from dissected muscle RNA-sequencing data from $ras1^{G12V};csk^{-/-}$ animals raised on HSD at Early Stage (Early), Mid Stage (Mid) and Late Stage (Late) compared to $ras1^{G12V};csk^{-/-}$ animals raised on CD. Data is displayed as cumulative enrichment score over the time course. Gene sets in purple are derived from Hallmark (H) datasets, gene sets in green are derived from Curated (C2) datasets and gene sets in pink are derived from Gene Ontology (C5) datasets. **g**, GSEA for the functional category 'MAPK Signalling'. **h, i**, phospho-ERK staining (red) of dissected body wall muscle from $ras1^{G12V};csk^{-/-}$ animals raised on CD (**h**) or HSD (**i**) with F-actin (green). Scale bar, 100 µm. **j,** F-actin (red) staining of dissected larval body wall muscle tissue from *mhc>btl.λ*, $rl^{RNAi/TRiP31524}$ third-instar larvae fed a HSD. Scale bar, 100 µm. **k**, Matching body wall muscle wasting quantification. **l**, Matching hemolymph proline quantification displayed as relative peak area ratio. Results are shown as mean ± SEM. Data from *n* = 4 biologically independent samples.

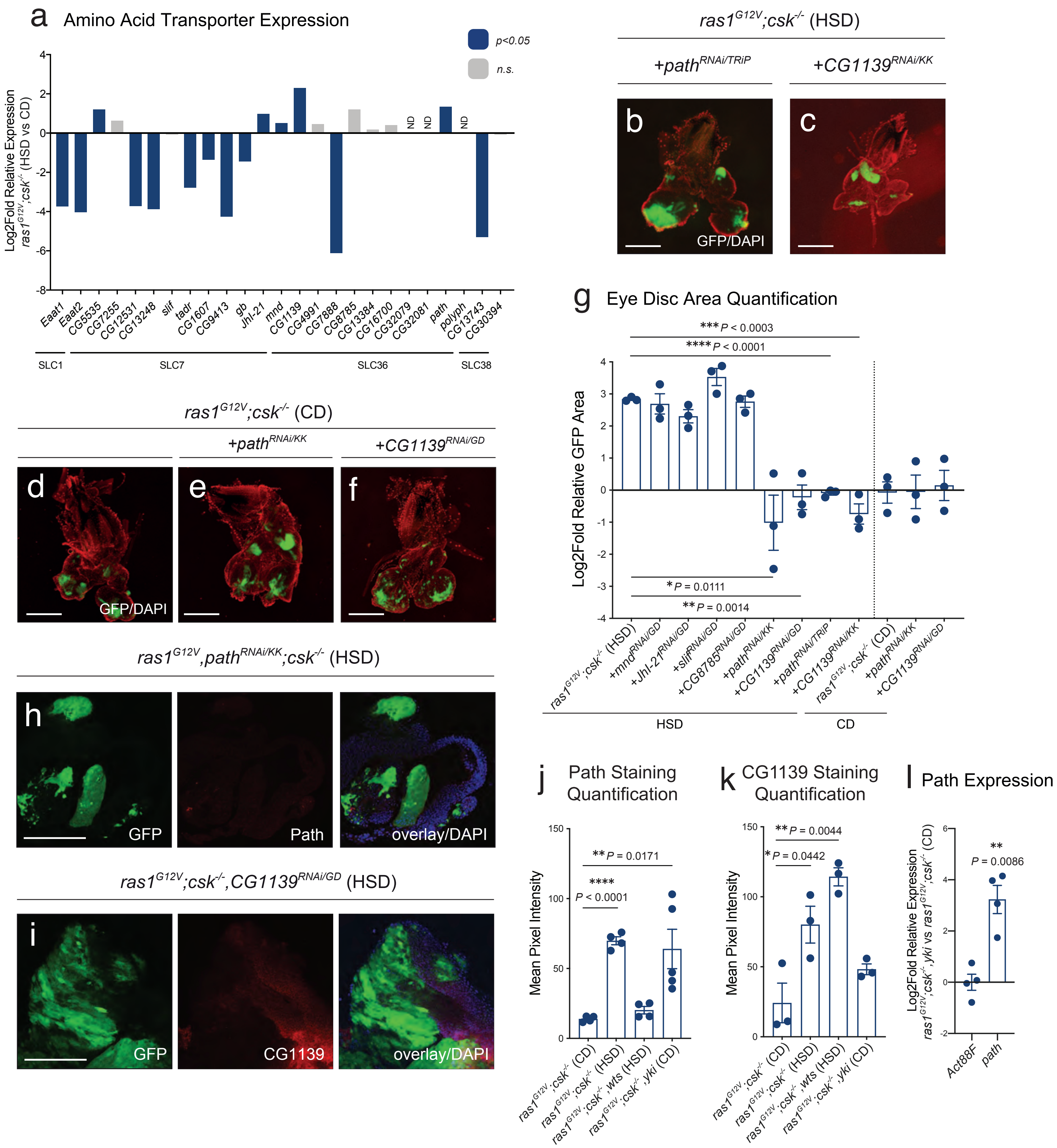

Supplementary Fig. 5

Supplementary Fig. 5. Identification of amino acid transporters elevated in HSD-enhanced Ras/Src-tumours.

a, Expression levels of amino acid transporters of the SLC1, SLC7, SLC36 and SLC38 families as measured by RNA-seq analysis. Gene expression levels are shown as the log2-relative fold change between *ras1*^{G12V}*;csk*$^{-/-}$ tumours in animals raised on HSD compared to animals raised on CD. Data from n = 4 (*ras1*^{G12V}*;csk*$^{-/-}$ CD) and n = 4 (*ras1*^{G12V}*;csk*$^{-/-}$ HSD) biologically independent samples. The two-sided P-value was calculated using Wald test and Benjamini-Hochberg method was applied for the adjusted P-value. Blue bars indicate significant cases where $P < 0.05$ (*Eaat1*, $P < 0.0001$; *Eaat2*, $P < 0.0001$; *CG5535*, $P < 0.0001$; *CG12531*, $P < 0.0001$; *CG13248*, $P < 0.0001$; *tadr*, $P < 0.0001$; *CG1607*, $P < 0.0001$; *CG9413*, $P < 0.0001$; *gb*, $P < 0.0001$; *JhI-21*, $P < 0.0001$; *mnd*, $P = 0.0047$, *CG1139* $P = 0.0225$; *CG7888*, $P < 0.0001$; *path*, $P < 0.0001$; *CG13743*, $P = 0.0010$). Grey bars indicate non-significant (*n.s.*) cases where $P > 0.05$. ND: not detected. **b, c,** Dissected eye epithelial tissue stained with DAPI (red) from animals raised on HSD with independent RNAi lines, *ras1*^{G12V} *,path*$^{RNAi/TRiP}$*;csk*$^{-/-}$ (**b**), or *ras1*^{G12V}*,CG1139*$^{RNAi/KK}$*;csk*$^{-/-}$ (**c**). Scale bar, 250 μm. **d-f**, Dissected eye epithelial tissue stained with DAPI (red) from *ras1*^{G12V}*;csk*$^{-/-}$ animals raised on CD (**d**), with *path*$^{RNAi/KK}$ (**e**)*,* and *CG1139*$^{RNAi/GD}$ (**f**). Scale bar, 250 μm. **g,** Eye disc area quantification. Results are shown as mean ± SEM. Data from n = 3 biologically independent samples. Data were analysed by two-tailed unpaired Student's t-test. Asterisks indicate statistically significant difference (*$P < 0.05$; **$P < 0.01$; ***$P < 0.001$; ****$P < 0.0001$). **h,** Anti-Path staining (red) of dissected tumour tissue from *ras1*^{G12V}*, path*$^{RNAi/KK}$*;csk*$^{-/-}$animals raised on HSD with DAPI (blue). Scale bar, 100 μm. **i**, Anti-CG1139 staining (red) of dissected tumour tissue from *ras1*^{G12V}*;csk*$^{-/-}$*,CG1139*$^{RNAi/GD}$ animals raised on HSD, with DAPI (blue). Scale bar, 100 μm. **j**, Path staining quantification. Results are shown as mean ± SEM. Data from n = 4 (*ras1*^{G12V}*;csk*$^{-/-}$ CD), n = 4 (*ras1*^{G12V}*;csk*$^{-/-}$ HSD), n = 4 (*ras1*^{G12V}*;csk*$^{-/-}$*,wts* CD), and n = 5 (*ras1*^{G12V}*;csk*$^{-/-}$*,yki* CD) biologically independent samples. Data were analysed by two-tailed unpaired Student's t-test. Asterisks indicate statistically significant difference (**$P < 0.01$; ****$P < 0.0001$). **k**, CG1139 staining quantification. Results are shown as mean ± SEM. Data from n =3 biologically independent samples. Data were analysed by two-tailed unpaired Student's t-test. Asterisks indicate statistically significant difference (*$P < 0.05$; **$P < 0.01$). **l,** Relative log2fold change of *path* in dissected tumour tissue from *ras1*^{G12V}*;csk*$^{-/-}$*,yki* animals raised on CD compared to *ras1*^{G12V}*;csk*$^{-/-}$ animals raised on CD, as determined by qPCR. Samples are normalised to *Act88F.* Results are shown as mean ± SEM. Data from n = 4 biologically independent samples. Data were analysed by two-tailed unpaired Student's t-test. Asterisks indicate statistically significant difference (**$P < 0.01$).

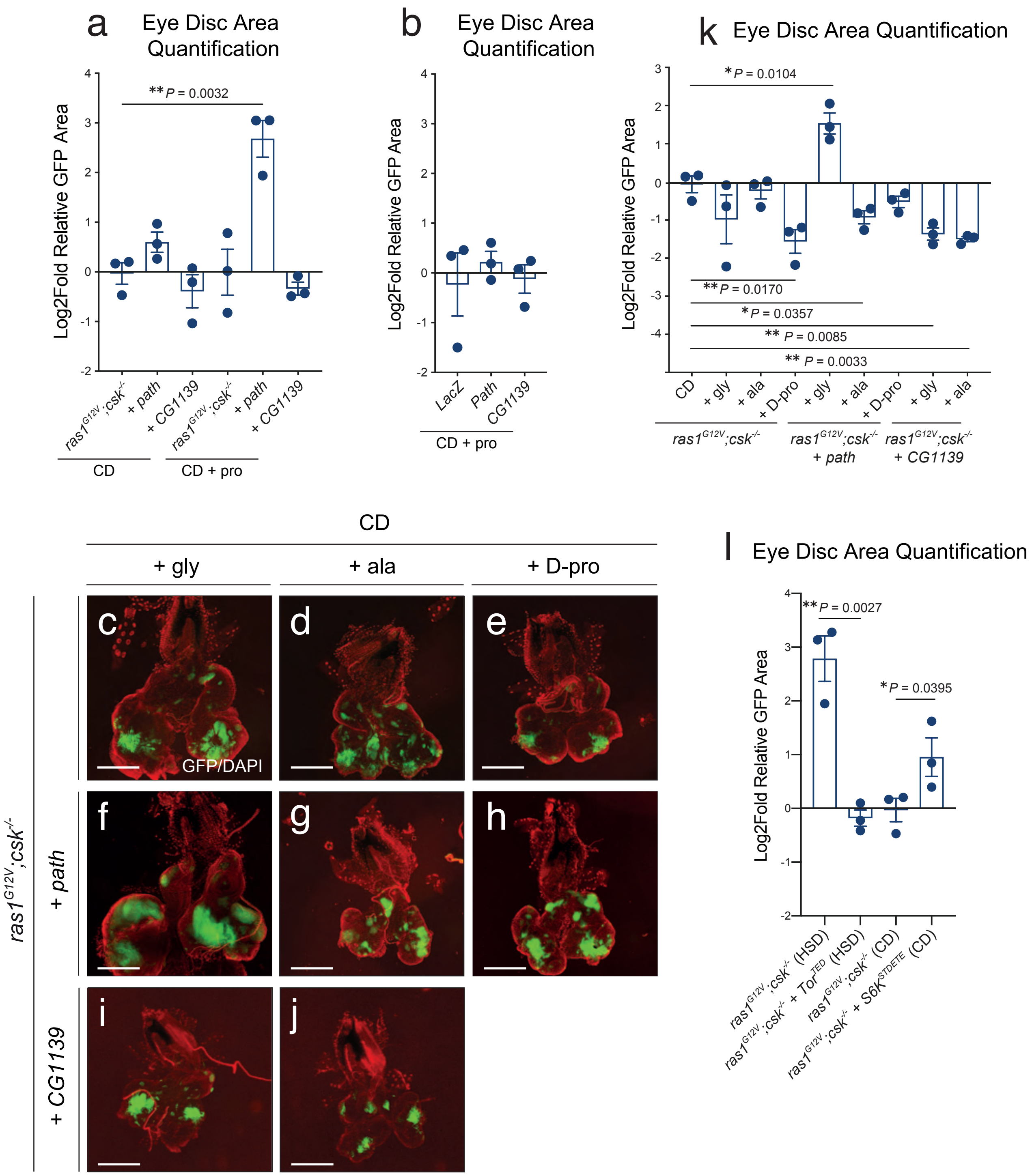

Supplementary Fig. 6

Supplementary Fig. 6. Amino acid specificity of Path-mediated Ras/Src-tumour growth.

a, Eye disc area quantification of Fig. 5a-f. Results are shown as mean ± SEM. Data from n = 3 biologically independent samples. Data were analysed by two-tailed unpaired Student's t-test. Asterisks indicate statistically significant difference (**$P < 0.01$). **b,** Eye disc area quantification of Fig. 5g-i. Results are shown as mean ± SEM. Data from n = 3 biologically independent samples. **c-e,** Dissected eye epithelial tissue stained with DAPI (red) from *ras1*^{G12V}*;csk*$^{-/-}$ animals raised on CD supplemented with 100 mM L-glycine (**c**), 100 mM L-alanine (**d**), and 100 mM D-proline (**e**). Scale bar, 250 µm. **f-h,** Dissected eye epithelial tissue stained with DAPI (red) from *ras1*^{G12V}*;csk*$^{-/-}$*,path* animals raised on CD supplemented with 100 mM L-glycine (**f**), 100 mM L-alanine (**g**), and 100 mM D-proline (**h**). Scale bar, 250 µm. **i, j,** Dissected eye epithelial tissue stained with DAPI (red) from *ras1*^{G12V}*;csk*$^{-/-}$*,CG1139* animals raised on CD supplemented with 100 mM L-glycine (**i**), and 100 mM L-alanine (**j**). Scale bar, 250 µm. **k,** Eye disc area quantification of Supplementary Fig. 6c-j. Results are shown as mean ± SEM. Data from n = 3 biologically independent samples. Data were analysed by two-tailed unpaired Student's t-test. Asterisks indicate statistically significant difference (*$P < 0.05$; **$P < 0.01$). **l,** Eye disc area quantification of Fig. 5k-n. Results are shown as mean ± SEM. Asterisks indicate statistically significant difference (*: p<0.05, ***; p<0.005). Results are shown as mean ± SEM. Data from n = 3 biologically independent samples. Data were analysed by two-tailed unpaired Student's t-test. Asterisks indicate statistically significant difference (*$P < 0.05$; **$P < 0.01$).

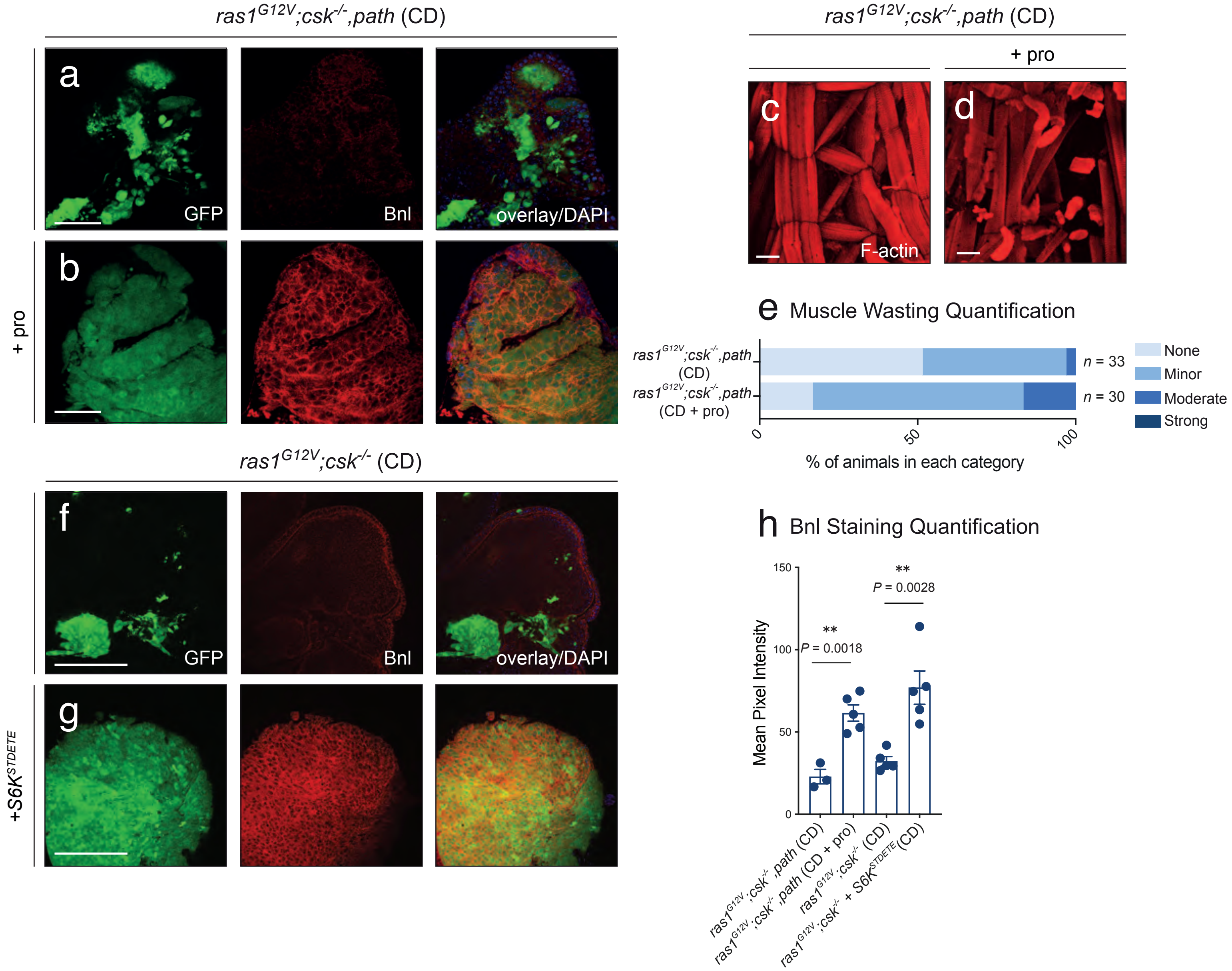

Supplementary Fig. 7

Supplementary Fig. 7. Proline-mediated Ras/Src tumour growth promotes Bnl-expression and muscle wasting.

a, b, Anti-Bnl staining (red) of dissected tumour tissue from *ras1*^{G12V}*;csk*$^{-/-}$*,path* animals raised on CD (**a**) or CD supplemented with 100mM L-proline (**b**) with DAPI (blue). Scale bar, 40 µm. **c, d,** F-actin (red) staining of dissected larval body wall muscle from *ras1*^{G12V}*;csk*$^{-/-}$*,path* animals raised on CD (**c**) or CD supplemented with 100mM L-proline (**d**). Scale bar, 100 µm. **e,** Matching body wall muscle wasting quantification. **f, g,** Anti-Bnl staining (red) of dissected tumour tissue from *ras1*^{G12V}*;csk*$^{-/-}$ (**f**) or *ras1*^{G12V}*;S6K*^{STDETE}*,csk*$^{-/-}$ (**g**) animals raised on CD. Scale bar, 40 µm. **h**, Matching Bnl staining quantification. Results are shown as mean ± SEM. Data from $n = 3$ (*ras1*^{G12V}*;csk*$^{-/-}$*,path* CD), $n = 5$ (*ras1*^{G12V}*;csk*$^{-/-}$*,path* CD + pro), $n = 5$ (*ras1*^{G12V}*;csk*$^{-/-}$ CD), and $n = 5$ (*ras1*^{G12V}*;S6K*^{STDETE}*,csk*$^{-/-}$ CD) biologically independent samples. Data were analysed by two-tailed unpaired Student's *t*-test. Asterisks indicate statistically significant difference (**$P < 0.01$).

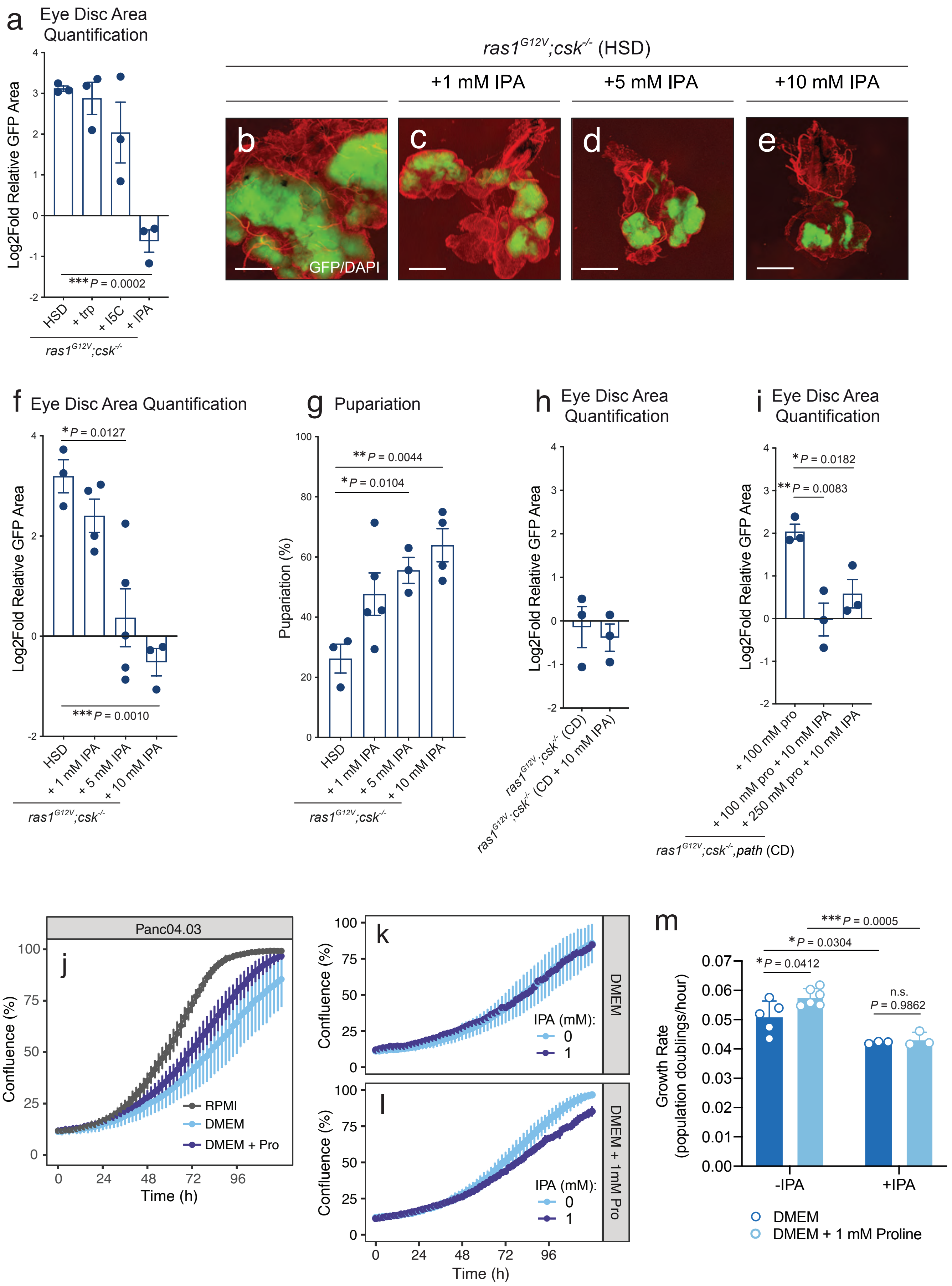

Supplementary Fig. 8

Supplementary Fig. 8. Targeting proline-mediated tumour growth.

a, Eye disc area quantification of Fig. 6a-d. Results are shown as mean ± SEM. Data from *n* = 3 biologically independent samples. Data were analysed by two-tailed unpaired Student's *t*-test. Asterisks indicate statistically significant difference (****P* < 0.001). **b-e,** Dissected eye epithelial tissue stained with DAPI (red) from *ras1*^{G12V}*;csk*$^{-/-}$ animals raised on HSD (**b**), HSD supplemented with 1 mM IPA (**c**), HSD supplemented with 5 mM IPA (**d**), and HSD supplemented with 10 mM IPA (**e**). Scale bar, 250 µm. **f,** Eye disc area quantification of Supplementary Fig. 8b-e. Results are shown as mean ± SEM. Data from *n* = 3 biologically independent samples. Data were analysed by two-tailed unpaired Student's *t*-test. Asterisks indicate statistically significant difference (**P* < 0.05; ****P* < 0.001). **g**, Pupariation percentage of *ras1*^{G12V}*;csk*$^{-/-}$ animals raised HSD supplemented with indicated concentrations of IPA. Results are shown as mean ± SEM. Data from total *n* = 47 from 3 independent experiments (*ras1*^{G12V}*;csk*$^{-/-}$ HSD), *n* = 133 from 5 independent experiments (*ras1*^{G12V}*;csk*$^{-/-}$ HSD + 1 mM IPA), *n* = 128 from 3 independent experiments (*ras1*^{G12V}*;csk*$^{-/-}$ HSD + 5 mM IPA), and *n* = 75 from 4 independent experiments (*ras1*^{G12V}*;csk*$^{-/-}$ HSD + 10 mM IPA). Data were analysed by two-tailed unpaired Student's *t*-test. Asterisks indicate statistically significant difference (**P* < 0.05; ***P* < 0.01). **h,** Eye disc area quantification of Fig. 6e, f. Results are shown as mean ± SEM. Data from *n* = 3 biologically independent samples. **i,** Eye disc area quantification of Fig. 6j-l. Results are shown as mean ± SEM. Data from *n* = 3 biologically independent samples. Data were analysed by two-tailed unpaired Student's *t*-test. Asterisks indicate statistically significant difference (**P* < 0.05; ***P* < 0.01). **j**, Growth curves of pancreatic cancer cell line Panc.04.03 in RPMI (grey, *n* = 6), DMEM (light blue, *n* = 6), and DMEM supplemented with 1 mM proline (dark blue, *n* = 6). Results are shown as mean ± SD. **k**, Growth curves of pancreatic cancer cell line Panc.04.03 in the presence (dark blue, *n* = 3) or absence (light blue, *n* = 6) of 1 mM IPA in DMEM. **l**, Growth curves of pancreatic cancer cell line Panc.04.03 in the presence (dark blue, *n* = 3) or absence (light blue, *n* = 6) of 1 mM IPA in DMEM supplemented with 1 mM proline. Results are shown as mean ± SD. **m**, Quantification of the growth rates of pancreatic cancer cell line Panc.04.03 in in DMEM (*n* = 6), DMEM + 1mM proline (*n* = 6), DMEM + IPA (*n* = 3), DMEM + 1mM proline + 1 mM IPA (*n* = 3). Results are shown as mean ± SD. Data were analysed by 2-way ANOVA with Tukey's multiple comparisons test (n.s. not significant; **P* < 0.05; ****P* < 0.001).

Gene	Forward (F) or Reverse (R)	Primers
Act88F	F	TCGGCTCGGACAGTGATAGA
	R	AAGGATGAGCACCGACAACC
bnl	F	CTCAAGTTCCGAGGCCCAAG
	R	GGATGGCTCTTTTTCGGAGCA
pyramus	F	GGTTGGAAAGAGAAACGCCAC
	R	ACGCAGCACTGGATTGTAGG
thisbe	F	TTAGTTGGCATGAGGGAGCTG
	R	CAGATCGACAACGGAACGGA
mnd	F	CTTTTTGGCCTTCCTGCGAC
	R	CAGCACACATATCATGGCAGC
JhI-21	F	TCTCGAGTGACAATGTGCGCTAT
	R	TGGTGGTCACTCCATTGGCA
slif	F	TTAGAGGCCAGCTAAACCGC
	R	CCAGAGATTGGTCATGGATTTTTGA
CG8785	F	AGAAACGATTGGATCGGGCA
	R	ATCTGCCATCTTTTGGACCGA
path	F	TGTTTGATTTGCGCGGCATT
	R	TTCGACCCGCTGTCCACTAT
CG1139	F	GGAAGCAGTGGGAGTGATCC
	R	ACAAGCCCAGCACTATGGAC
ImpL2	F	AAGAGCCGTGGACCTGGTA
	R	TTGGTGAACTTGAGCCAGTCG
upd1	F	AACTGGATCGACTATCGCAAC
	R	CTATGGCCGAGTCCTGGCTAC
upd2	F	TACAAGTTCCTGCCGAACATG
	R	ATGTGGCGGTACCAAGTCTTT
upd3	F	ACAAGTGGCGATTCTATAAGG
	R	ATGTTGCGCATGTACGTGAAG
Pvf1	F	GCGCAGCATCATGAAATCAACCG
	R	TGCACGCGGGCATATAGTAGTAG
Pvf2	F	TCAGCGACGAAACGTGCAAGA
	R	TTTGAATGCGGCGTCGTTCC
Pvf3	F	AGCCAAATTTGTGCCGCCAAG
	R	CTGCGATGCTTACTGCTCTTCACG
eiger	F	GATGGTCTGGATTCCATTGC
	R	TAGTCTGCGCCAACATCATC

Supplementary Table 1. List of primers used in this study.

Compound	Mass [m/z]	Fragment [*m/z*]	Formula [M]	Start [min]	End [min]	NCE
L-Proline	116.07061	70.0651	$C_5H_9NO_2$	2.00	4.00	60
$^{13}C_5{}^{15}N$-L-Proline	122.08441	75.0757	$[^{13}C]_5H_9[^{15}N]O_2$	2.00	4.00	60
L-Proline-2,5,5-d3	119.08944	73.0841	$C_5D_3H_6NO_2$	2.00	4.00	50

Supplementary Table 2. Inclusion list used for the PRM experiment.
