# Supplementary material for: Systemic muscle wasting and coordinated tumour response drive tumourigenesis
Source: Nat Commun. 2020 Sep 16;11:4653. doi: 10.1038/s41467-020-18502-9 (PMC7495438; doi:10.1038/s41467-020-18502-9)
Supplement: Supplementary file 2 — Reporting Summary [file 41467_2020_18502_MOESM2_ESM.pdf]

nature research

Corresponding author(s): Susumu Hirabayashi
Last updated by author(s): Aug 4, 2020

Reporting Summary

Nature Research wishes to improve the reproducibility of the work that we publish. This form provides structure for consistency and transparency in reporting. For further information on Nature Research policies, see Authors & Referees and the Editorial Policy Checklist .

Statistics

For all statistical analyses, confirm that the following items are present in the figure legend, table legend, main text, or Methods section.

n/a	Confirmed	
☐	☒	The exact sample size (n) for each experimental group/condition, given as a discrete number and unit of measurement
☐	☒	A statement on whether measurements were taken from distinct samples or whether the same sample was measured repeatedly
☐	☒	The statistical test(s) used AND whether they are one- or two-sided<br>*Only common tests should be described solely by name; describe more complex techniques in the Methods section.*
☒	☐	A description of all covariates tested
☐	☒	A description of any assumptions or corrections, such as tests of normality and adjustment for multiple comparisons
☐	☒	A full description of the statistical parameters including central tendency (e.g. means) or other basic estimates (e.g. regression coefficient) AND variation (e.g. standard deviation) or associated estimates of uncertainty (e.g. confidence intervals)
☐	☒	For null hypothesis testing, the test statistic (e.g. F, t, r) with confidence intervals, effect sizes, degrees of freedom and P value noted<br>*Give P values as exact values whenever suitable.*
☒	☐	For Bayesian analysis, information on the choice of priors and Markov chain Monte Carlo settings
☒	☐	For hierarchical and complex designs, identification of the appropriate level for tests and full reporting of outcomes
☒	☐	Estimates of effect sizes (e.g. Cohen's d, Pearson's r), indicating how they were calculated

Our web collection on statistics for biologists contains articles on many of the points above.

Software and code

Policy information about availability of computer code

Data collection	Microscopy: Confocal images were taken with Leica SP5 II confocal microscope and Leica Application Suite Advanced Fluorescence software ver. 4.6.1 (Leica Microsystems).<br>Larval Video Tracking: Video plate recordings were carried out with use of a Teledyne DALSA Genie Nano Camera (G3-GM11-M2420) using Gecko GigE Video Recorder software (v2.0.3.1; www.visionexperts.co.uk).<br>Quantitative RT-PCR: 7900HT Real-Time PCR system with Applied Biosystems Software (SDS v2.4).<br>RNA-sequencing: Illumina Hiseq 2500<br>Metabolomics: Agilent CE-TOFMS Machine (CE-TOFMS) and Agilent 6460 TripleQuad LC/MS Machine (CE-QqQMS)(Agilent Technologies)
Data analysis	Image Processing: Adobe Photoshop CC software (19.1.6)<br>Larval Video Tracking: Previously published Tierpsy Tracker code (Reference 12: Javer et al. Nature Methods. 2018)<br>Quantitative RT-PCR: Data analysis was carried out in Microsoft Excel (16.16.4)<br>RNA-sequencing: TopHat2 (v2.0.11) for genome alignment and transcript annotation. TrimGalore (v0.4.4) (http://www.bioinformatics.babraham.ac.uk/projects/trim_galore/) for trimming adaptor and low quality reads. The R/Bioconductor package Rsubread (v1.32.1) function featureCounts for gene-based read counts. The Bioconductor package DESeq2 (v2_1.19.52) for differential expression analysis.<br>Metabolomics: MasterHands v.2.17.1.11 (developed at Keio University). MassHunter Quantitative Analysis B.06.00 (Agilent Technologies).<br>Immunostaining Quantification: ImageJ software (ver. 2.0.0-rc-43).<br>Cell Growth Rate Calculation: Growthcurver package in R (ver.0.3.0)

For manuscripts utilizing custom algorithms or software that are central to the research but not yet described in published literature, software must be made available to editors/reviewers. We strongly encourage code deposition in a community repository (e.g. GitHub). See the Nature Research guidelines for submitting code & software for further information.

Data

Policy information about availability of data

All manuscripts must include a data availability statement. This statement should provide the following information, where applicable:

- Accession codes, unique identifiers, or web links for publicly available datasets
- A list of figures that have associated raw data
- A description of any restrictions on data availability

All raw RNA-sequencing data that support the findings of this study have been deposited in the GEO repository with the accession codes GSE139958, available at https://www.ncbi.nlm.nih.gov/geo/query/acc.cgi?acc=GSE139958. Other data that support the study are available from the corresponding author upon reasonable request.

Field-specific reporting

Please select the one below that is the best fit for your research. If you are not sure, read the appropriate sections before making your selection.

[x] Life sciences [ ] Behavioural & social sciences [ ] Ecological, evolutionary & environmental sciences

For a reference copy of the document with all sections, see nature.com/documents/nr-reporting-summary-flat.pdf

Life sciences study design

All studies must disclose on these points even when the disclosure is negative.

Sample size	Given the use of Drosophila, sample size was typically not a limiting factor. Sample sizes were chosen empirically based on the variability of each scored phenotype. For pooled tissue samples collected for RNA-sequencing or metabolomics experiments, sample size was determined as the value required to ensure enough material was available given the technical limitations of the technique to be used.
Data exclusions	No data was excluded.
Replication	Each experiment was conducted with multiple biological replicates , with numbers indicated in each figure or figure legend.
Randomization	For experimental crosses, 15-25 virgin female flies were mated with 6-10 males of the appropriate genotype per independent vial and were randomised whenever possible. All offspring larvae were collected at the appropriate time point for experimenting. Larvae of the appropriate genotype were pre-selected and animals were chosen from this group at random up to the determined sample size.
Blinding	The investigators were not blinded during the experiments.

Reporting for specific materials, systems and methods

We require information from authors about some types of materials, experimental systems and methods used in many studies. Here, indicate whether each material, system or method listed is relevant to your study. If you are not sure if a list item applies to your research, read the appropriate section before selecting a response.

Materials & experimental systems

n/a	Involved in the study	
[ ]	[x]	Antibodies
[ ]	[x]	Eukaryotic cell lines
[x]	[ ]	Palaeontology
[ ]	[x]	Animals and other organisms
[x]	[ ]	Human research participants
[x]	[ ]	Clinical data

Methods

n/a	Involved in the study	
[x]	[ ]	ChIP-seq
[x]	[ ]	Flow cytometry
[x]	[ ]	MRI-based neuroimaging

Antibodies

Antibodies used	Primary antibodies used were: rat anti-myosin (ab51098; Abcam; 1:100) and mouse anti-phospho-ERK (M-8159; Sigma; 1:50). Polyclonal Rabbit anti-CG1139 (1:200) was purified by New England Peptide Inc., MA, USA. An amino acid sequence 26-40 was selected as the epitope. The following primary antibodies were kindly gifted to us: rat anti-bnl (M. Krasnow; 1:50), guinea pig anti-Path (J. Parrish; 1:200), and rabbit anti-phosho Drosophila S6 (pS6) (A. Teleman; 1:500). Secondary Alexa-488 and Alexa-568 conjugated antibodies were from Molecular Probes (1:200). Secondary antibodies used were: Alexa Fluor 488 and 568 conjugated anti-rat antibody (1:200; Thermo Fisher Scientific, A-11006, A-11077), Alexa Fluor 568 conjugated anti-mouse antibody (1:200; Thermo Fisher Scientific, A-11031), Alexa Fluor 568 conjugated anti-rabbit antibody (1:200; Thermo Fisher Scientific, A-11036) and Alexa-568 conjugated anti-guinea pig antibody (1:200; Thermo Fisher Scientific, A-11075).
Validation	Rat anti-myosin (ab51098; Abcam) has been validated by the manufacturer to react with subfragment 2 of Drosophila myosin heavy chain, and suitable for immunocytochemistry. Mouse anti-phospho ERK (M-8159; Sigma) has been validated by the manufacturer to react specifically with the diphosphorylated form of MAP kinase in Drosophila and other species, and suitable for immunocytochemistry. We validated polyclonal Rabbit anti-CG1139 (New England Peptide Inc.) by CG1139 knock down and immunostaining in Ras/Src - HSD tumour background (Fig. 4n and Supplementary Fig. 5i). Rat anti-bnl (M. Krasnow) has been previously validated in Drosophila with citations including: Mukherjee et al. G3. 2012. We validated guinea pig anti-path (J.Parrish) by Path knock down and immunostaining in Ras/Src - HSD tumour background (Figure 4j and Supplementary Fig. 5h). Rabbit anti-phospho Drosophila S6 (pS6) has been previously validated in Drosophila (Romero-Pozuelo et al. Developmental Cell. 2017).

Eukaryotic cell lines

Policy information about cell lines

Cell line source(s)	Panc04.03 (ATCC CRL-2555) were obtained from the American Type Culture Collection (ATCC, Manassas, VA, USA).
Authentication	Panc04.03 (ATCC CRL-2555) were authenticated at ATCC.
Mycoplasma contamination	Cell lines were tested for mycoplasma contamination using the MycoAlert Mycoplasma detection kit (Lonza) and found to be mycoplasma-free.
Commonly misidentified lines (See ICLAC register)	*Name any commonly misidentified cell lines used in the study and provide a rationale for their use.*

Animals and other organisms

Policy information about studies involving animals; ARRIVE guidelines recommended for reporting animal research

Laboratory animals	The model organism Drosophila melanogaster was the only species used for this study. Fly lines were obtained from the following sources: UAS-lacZ (BDSC: 8529), ecd1 (BDSC: 218), UAS-bnlRNAi/TRiP (BDSC: 34572), UAS-ImpL2RNAi/TRiP (BDSC: 55855), UAS-Pvf2RNAi/TRiP (BDSC: 61955), UAS-bnl (BDSC: 64231), mhc-gal4 (BDSC: 55133), UAS-btl.lambda (BDSC: 29045), UAS-rlSEM (BDSC: 59006), and UAS-rlRNAi/TRiP (BDSC: 31387), UAS-rlRNAi/TRiP (BDSC: 31524), UAS-pathRNAi/TRiP (BDSC: 64029), UAS-wts (BDSC: 44258), UAS-Tor.TED (BDSC: 7013), and UAS-S6K.STDETE (BDSC: 6914) flies were obtained from the Bloomington Drosophila Stock Center. UAS-bnlRNAi/GD (VDRC: 5730), UAS-ImpL2RNAi/GD (VDRC: 30930), UAS-Pvf1RNAi/GD (VDRC: 6173), UAS-Pvf1RNAi/KK (VDRC: 102699), UAS-Pvf2RNAi/KK (VDRC: 102072), UAS-btlRNAi/GD (VDRC: 950), UAS-btlRNAi/KK (VDRC: 110277), UAS-mndRNAi/GD (VDRC: 42485), UAS-slifRNAi/GD (VDRC: 45590), UAS-Jhl-21RNAi/GD (VDRC: 45193), UAS-CG8785RNAi/GD (VDRC: 4650), UAS-pathRNAi/KK (VDRC: 100519), UAS-CG1139RNAi/GD (VDRC: 8907), and UAS-CG1139RNAi/KK (VDRC: 102363) flies were obtained from the Vienna Drosophila Resource Center. The following stocks were kindly provided to us: FRT82B, csk.Q156Stop by A. O'Reilly and M. Simon, ey(3.5)-FLP1 and UAS-ras1.G12V by G. Halder, FB-gal4 by R. Kühnlein, UAS-pathA by J. Parrish, UAS-CG1139 by D. Goberdhan, and UAS-Yki.V5 by K. Irvine. A complete list of recombinant fly lines generated for experimental crosses are listed in the materials and methods section of the manuscript.
Wild animals	This study does not involve wild animals.
Field-collected samples	This study does not involve animals collected from the field.
Ethics oversight	No ethical approval was required to work with Drosophila melanogaster.

Note that full information on the approval of the study protocol must also be provided in the manuscript.
